# Aberrantly High FBXO31 Impairs Oocyte Quality in Premature Ovarian Insufficiency

**DOI:** 10.14336/AD.2023.0809

**Published:** 2024-04-01

**Authors:** Feiyan Zhao, Long Yan, Xuehan Zhao, Jiaqi Wu, Ying Fang, Zhimin Xin, Hongmei Wang, Xiaokui Yang

**Affiliations:** ^1^Department of Human Reproductive Medicine, Beijing Obstetrics and Gynecology Hospital, Capital Medical University, Beijing, China.; ^2^Beijing Maternal and Child Health Care Hospital, Beijing, China.; ^3^State Key Laboratory of Stem Cell and Reproductive Biology, Institute of Zoology, Chinese Academy of Sciences, Beijing, China.; ^4^University of Chinese Academy of Sciences, Beijing, China.; ^5^Institute for Stem Cell and Regeneration, Chinese Academy of Sciences, Beijing, China.; ^6^Beijing Institute for Stem Cell and Regenerative Medicine, Beijing, China.; ^7^Department of Obstetrics and Gynecology, Beijing Chao-Yang Hospital, Capital Medical University, Beijing, China.

**Keywords:** cell apoptosis, FBXO31, oocyte, premature ovarian insufficiency, reactive oxygen species

## Abstract

Premature ovarian insufficiency (POI), which is defined as loss of ovarian function that occurs before the age of 40, causes menstrual disturbances, infertility, and diverse health problems in females. Despite the limited understanding of the molecular basis underlying POI pathology, we had previously demonstrated that the cooperation of miR-106a and FBXO31 plays a pivotal role in diminished ovarian reserve (DOR), with FBXO31 serving as a putative target of miR-106a. In this study, we found that FBXO31 is aberrantly expressed in granulosa cells of POI patients, leading to accumulated reactive oxygen species (ROS) and cell apoptosis via the p53/ROS pathway. Furthermore, our results demonstrated that high levels of FBXO31 in mouse ovaries impair oocyte quality. Our study revealed that FBXO31 may serve as a novel indicator and play a significant role in the etiology of POI.

## INTRODUCTION

Premature ovarian insufficiency (POI) occurs in more than 1% of females under the age of 40, which results in menstrual disturbances, infertility, as well as diverse health problems [1]. Although hormone replacement therapy has ameliorated the health risks and improved the life quality of POI patients, medical treatment has so far provided limited help to the rehabilitation of ovarian function [2, 3]. The major etiologies of POI include chromosomal abnormalities and gene mutations, autoimmune factors, and iatrogenic causes, such as surgery, chemotherapy, and radiation therapy [2]. In recent studies, more than 50 genes, including stromal antigen 3 (STAG3), synaptonemal complex central element 1 (SYCE1), mini-chromosome maintenance complex component 8 and 9 (MCM8, MCM9), and the ATP-dependent DNA helicase homolog (HFM1), have been identified as specific pathogenic genes related to POI [2, 4]. Nevertheless, the mechanisms for POI still need to be explored.

POI generally arises from the dysfunction or depletion of ovarian follicles [5]. Numerous studies have demonstrated the tight connections between follicular atresia and granulosa cell (GC) apoptosis mediated by various cell death pathways [6, 7]. The absence of brain and reproductive expression (BRE) increases the apoptosis of granulosa cells and induces follicular atresia [8]. Wang et al. have pointed out that the decreased expression of lncRNA HCP5 in biochemical POI leads to dysfunctional GCs by directing MSH5 transcription and DNA damage repair [9]. Accumulating evidence has shown that microRNA, acetylation, ubiquitination, and oxidative stress are also deeply involved in the initiation of GC apoptosis among atretic follicles, and even subfertility [10-15]. Additionally, follicular atresia can be attributed to abnormal oocyte development, as women with POI suffer from a physiological decline in the quantity and quality of oocytes. A number of oocyte development-related genes, STAG3, SMC1β, REC8, SYCE1 PSMC3IP, SPIDR, and HFM1, have been identified to be associated with the onset of POI [1].

F-box only protein 31 (FBXO31), a member of the F-box protein family, acts as a substrate recognizing the subunit of Skp1-Cullin-F-box (SCF) ubiquitin E3 ligase complex [16]. Being implicated to participate in the ubiquitin-dependent proteasomal degradation pathways and the signal transductions of cell cycle and immune regulation [17, 18], FBXO31 has elucidated its dual role in tumorigenesis. It functions as a tumor suppressor in gastric and breast cancer by inhibiting epithelial-mesenchymal transition (EMT) and cell proliferation [19, 20]. Also, it acts as an oncogene to promote cell growth, migration, and invasion in lung cancer [17]. Typically, FBXO31 degrades cyclin D1 upon genotoxic stress to arrest cells at the G1 phase and eliminates MDM2 for robust stabilization of p53. This, in turn, initiates the appropriate DNA damage response [18, 21]. Intriguingly, a recent report has indicated that FBXO31 could inactivate the MDM2/p53 axis through the phospholipid inositol 3-kinase (PI3K)/protein kinase B (AKT) signaling pathway, thus inhibiting cell viability, invasion, migration, EMT, and inducing apoptosis in SiHa cells with FBXO31 overexpression [22]. Furthermore, it has been demonstrated that miRNA clusters containing miR-29c, miR-210, miR-93, and miR-106 can interact with FBXO31 in managing the chemoresistance and cell proliferation of esophageal and breast cancer [20, 23, 24]. We have previously identified that downregulated miR-106a contributes to the pathogenesis of diminished ovarian reserve (DOR) by reducing granulosa cell viability and increasing apoptosis [25]. Given the predominant role of FBXO31 in regulating cell survival, we reasonably infer its involvement in follicular atresia and the occurrence of ovarian disorders. However, further research is needed to determine the precise functions and molecular mechanisms of FBXO31 in the pathogenesis of POI.

In this study, we detected aberrantly high FBXO31 in granulosa cells from POI patients and demonstrated its ability to upregulate p53 through MDM2. We also found that FBXO31 could increase apoptosis via the p53/ROS signaling pathway in granulosa cells. Furthermore, we uncovered that augmented ROS levels induced by FBXO31 overexpression across granulosa cells and oocytes pose a detrimental effect on oocytes *in vivo*. Taken together, we pinpointed FBXO31 as a novel marker that hastens the progression of POI and elucidated its regulatory effects on inducing cell apoptosis and ovarian damage.

## MATERIALS AND METHODS

### Study population

The study participants included 24 POI women (POI group) and 25 normal ovarian reserve women (control group) undertaking IVF-ET at the department of Human Reproductive Medicine, Beijing Obstetrics and Gynecology Hospital from January 1, 2019, to June 30, 2021. Women in the control group sought infertility treatment due to tubal obstruction or male factors, with regular menstrual cycles and normal endocrine profiles. The diagnostic criteria of POI were as follows: 1) under 40 years old; 2) at least one year of amenorrhea; 3) two or more instances of FSH > 25 IU/L (i.e., two analyses at an interval of one month or more); and 4) serum estradiol < 20 pg/ml [26]. The Ethics Committee of the Beijing Obstetrics and Gynecology Hospital, Capital Medical University approved the use of primary human granulosa cells (hGCs) for the following experiments (Code: 2018-KY-066). Informed written consent was obtained from all participants included in the study.

### Primary hGC samples collection and cell culture

Primary hGCs were isolated from follicular fluid obtained at oocyte retrieval as previously described [25]. Briefly, follicular fluid was centrifuged at 400 × g for 10 minutes (min), and the layer of granulosa cells with red blood cell pellet was resuspended. After shaking at 200 rpm for 20 min at 37°C, the cell suspension was layered on 8.0 mL Ficoll-Paque Plus (GE Healthcare, USA) and centrifuged at 600 × g for 20 min. Granulosa cells at the interface were harvested and washed three times with 10 mL Dulbecco’s modified Eagle medium/nutrient mixture F-12 Ham (DMEM/F-12, Gibco, USA) supplemented with 10% fetal bovine serum (FBS, Gibco), 1% penicillin-streptomycin (5,000 U/mL, Gibco), and 1×GlutaMAX (Invitrogen, China).

Primary hGCs, which were cultured in DMEM/F-12 medium supplemented with 10% FBS, 1% penicillin-streptomycin, and 1×GlutaMAX, were transfected on the third day of cell culture. Human granulosa-like tumor cell line (KGN) cells were purchased from Beijing Beina Chuanglian Biotechnology Institute and cultured in DMEM/F-12 medium supplemented with 10% FBS and 1% penicillin-streptomycin. The cells were routinely incubated at 37°C in a humid atmosphere containing 5% CO_2_.

### RNA sequencing and bioinformatics analysis

Total RNA was extracted from three FBXO31-overexpressed KGN cell samples and three vector control samples using Fast Pure Cell Tissue Total RNA Isolation Kit V2 (RC112-01, Vazyme, China). The amount and purity of RNA were quantified using a NanoDrop ND-1000 (NanoDrop, USA). RNA samples were submitted for paired-end RNA-seq on an Illumina HiSeq following the vendor’s recommended protocol.

Raw data (raw reads) of fastq format were first processed through in-house perl scripts. In this step, clean data (clean reads) were obtained by removing reads containing adapter, reads containing ploy-N and low-quality reads. At the same time, Q20, Q30, GC-content, and sequence duplication levels of clean data were calculated. All the downstream analyses were based on clean data with high quality. The adaptor sequences and low-quality sequence reads were removed from the data sets. Raw sequences were transformed into clean reads after data processing. These clean reads were then mapped to the reference genome sequence. Only reads with a perfect match or one mismatch were further analyzed and annotated based on the reference genome. Hisat2 tools soft was used to map with the reference genome (homo sapiens. GRCh38).

Differential expression analysis of two groups was performed using the DESeq2 R package (1.26.0). DESeq2 provides statistical routines for determining differential expression in digital gene expression data using a model based on the negative binomial distribution. The resulting *p*-values were adjusted using Benjamini and Hochberg’s approach for controlling the false discovery rate (FDR). Genes with FDR < 0.05 and |log2(fold change)| ≥ 1 found by DESeq2 were assigned as differentially expressed.

Gene Ontology (GO) enrichment analysis of differentially expressed genes (DEGs) was implemented by the GOseq R packages based on Wallenius non-central hyper-geometric distribution, which can adjust gene length bias in DEGs. Kyoto Encyclopedia of Genes and Genomes (KEGG) is a database resource for understanding high-level functions and utilities of the biological system from molecular-level information, especially large-scale molecular datasets generated by genome sequencing (http://www.genome.jp/kegg/). We used KOBAS software to test the statistical enrichment of DEGs in KEGG pathways.

### qRT-PCR

Total RNA was extracted from cells using Fast Pure Cell Tissue Total RNA Isolation Kit V2 (RC112-01, Vazyme). The mRNA was reverse-transcribed into cDNA using First-strand cDNA Synthesis Mix With gDNA Remover (F0201, Lablead, China). The target mRNA was measured using qRT-PCR by 2× Realab Green PCR Fast mixture (R0201-02, Lablead), and gene expression was normalized to calculated GAPDH Ct values. The primer sequences used are listed in Supplementary Table 1.

### FBXO31 3’-UTR vector construction and luciferase reporter assay

The targets of miR-106a-5p were predicted by MiRanda and TargetScan (http://www.targetscan.org/vert_72/), which were simultaneously used to predict the candidate binding sites of FBXO31. PGL3-CMV-LUC-FBXO31 3’UTR (75-81)-MUT plasmid (FBXO31 3’UTR mut1), PGL3-CMV-LUC-FBXO31 3’UTR (145-151)-MUT plasmid (FBXO31 3’UTR mut2), and PGL3-CMV-LUC-FBXO31 3’UTR (75-81) (145-151)-MUT plasmid (FBXO31 3’UTR mut3) for dual luciferase miRNA target expression were constructed in Genomeditech (China). 293T cells were seeded into the 24-well plate (1.5 × 10^5^/well). When the cells reached a 70% confluence, the wild type (WT) and the mutated 3′UTR plasmids were transfected using the Lipofectamine 3000 reagent (Invitrogen) with or without miR-106a mimic. After 24 hours (h), cells were lysed, and the luciferase activity was measured in triplicate using the Dual Luciferase Assay System (Genomeditech).

### Transfection of small interfering RNA

Short interfering RNAs (siRNAs) were purchased from Ribobio (Guangzhou, China). hGCs (2 × 10^5^) were seeded into six-well plates, cultured overnight, and transfected with siRNAs using the Lipofectamine 3000 Reagent according to the manufacturer’s protocol. After incubation for 48 h, the efficiency of silencing was confirmed by qRT-PCR and Western blotting (WB). The specific sequences of the target genes were as follows: siRNA-FBXO31#1, 5’-GCCTGGAGATTGTGATGCT-3’; siRNA-FBXO31#2, 5’-GATGACCCTATGAGATTC A-3’.

### Establishment of stably infected KGN cells with exogenous FBXO31 and control lentivirus

KGN cells were cultured to achieve a 30%-40% confluence, and transfected with PLVX-CMV-3×Flag-FBXO31-PGK-Puro WT (FBXO31 OE), PLVX-CMV-3 × Flag-FBXO31(g.153-327del)-PGK-Puro MT (FBXO31 △F OE), or PLVX-CMV-eGFP-PGK-Puro (Vector) lentivirus at a multiplicity of infection (MOI) was approximately five in the presence of 2 μg/ml of polybrene (Genomeditech). Two days following transfection, cells were enriched with puromycin (2 mg/ml) for seven days. Stable cell lines were examined for FBXO31 expression by qRT-PCR, WB, and green fluorescent protein (GFP) expression.

### Western blotting and cycloheximide chase assay

Western blotting was performed as described previously [27]. Cells treated with 2 μM nutlin-3a (N6287, Sigma, USA) for 12 h, or 5 μM MG132 (M1902, Abmole Bioscience, China) for 8 h, or 10 mM N-Acetylcysteine (NAC, M5385, Abmole Bioscience) for 24 h were lysed in ice-cold RIPA lysis buffer (P0013B, Beyotime, China) containing a protease inhibitor cocktail (Roche, Switzerland) and phosphatase inhibitor (Roche). Then, 25 μg protein was subjected to sodium dodecyl sulfate-polyacrylamide gel electrophoresis (SDS-PAGE) before being electrotransferred onto PVDF membranes. After blocking with 5% skim milk for 1 h, the membranes were incubated with primary antibodies against FBXO31 (1:3500; ab86137; Abcam, UK), p53 (1:200; sc-126; Santa Cruz Biotech, USA), p-p53 (1:1000; #9286P; Cell Signaling Technology, USA), MDM2 (1:2000; #86934S; Cell Signaling Technology), caspase-3 (1:1000; #9662S; Cell Signaling Technology), cleaved caspase-3 (1:1000; #9661S; Cell Signaling Technology), Bax (1:1000; K0022889P; Solarbio, China), Bcl-2 (1:1000; #15071S; Cell Signaling Technology), PGC1α (1:2000; ab54481; Abcam), SOD1 (1:3000; K106503P; Solarbio), SOD2 (1:1000; K106586P; Solarbio), UCP2 (1:500; K009212P; Solarbio), or GAPDH (1:2000; ab22556; Abcam) overnight at 4°C, followed by incubation with corresponding horseradish peroxidase (HRP)-conjugated secondary antibodies (Zhongshan Golden Bridge Biotechnology Co., Ltd., China) for 1 h at room temperature (RT). Signals were developed using the enhanced chemiluminescence system (Thermo Fisher Scientific, USA). Densitometric quantification was performed using Scion Image software (Scion Corp., USA).

The cells were treated with cycloheximide (100 μg/ml) for indicated time points (0 h, 6 h, and 12 h). Cells were then harvested, washed with ice-cold PBS, and lysed as described above. Cell lysates were subjected to WB, with GAPDH used as loading controls. Band intensity was measured using ImageJ software. Protein at the 0-hour time point was taken as 100%, and the percentage of remaining protein was calculated with respect to 0-hour.

### Immunofluorescence, mito-tracker and Annexin V-mCherry staining

Slides of stably infected KGN cells with exogenous FBXO31 were fixed in 4% paraformaldehyde (PFA) for 30 min at RT, and then permeabilized with 0.25% Triton X-100 for 20 min. After washing with PBS, the cells were blocked with 2% BSA for 30 min, and then incubated with primary antibodies including p53 (1:100; sc-126; Santa Cruz Biotech), Bax (1:100; #5023; Cell Signaling Technology), or Bcl-2 (1:100; #15071S; Cell Signaling Technology) for 30 min at RT. After washing, the cells were incubated with corresponding secondary antibodies (1:100, Invitrogen) for 1 h in the dark at RT. Mitochondria were stained with 200 nM Mito-Tracker-Red^®^ (Solarbio) for 30 min at 37°C, while the nuclei were counterstained with 4’,6-diamidino-2-phenylindole (DAPI, 1:1000; Sigma) for 10 min at RT. Finally, the slides were washed and mounted with an anti-fade medium (Beyotime).

Oocytes were fixed in 4% PFA with 0.1% BSA for 30 min at 37°C and washed three times with 0.1% BSA. Next, they were permeabilized in 0.5% Triton X-100 with 0.1% BSA for 30 min at RT, blocked with 5% BSA for 1 h, and incubated overnight at 4°C with primary antibodies γ-H2A.X (1:100; ab2893; Abcam) and α-tubulin (1:200; ab52866; Abcam). After washing three times in 0.1% BSA, the oocytes were incubated with corresponding secondary antibodies with DAPI for 1 h in the dark at RT. Finally, oocytes were transferred into a four-chamber glass bottom dish and covered with a thin layer of mineral oil. For Annexin V-mCherry staining, oocytes were collected and washed three times with 0.1% BSA using a mouth pipette, and then incubated in the binding buffer and Annexin V-mCherry (Beyotime) together with DAPI for 30 min in the dark at RT. Finally, oocytes were washed three times, transferred, and covered with a thin layer of mineral oil.

Paraffin-embedded sections were deparaffinized, rehydrated, and boiled in citrate buffer for antigen retrieval as previously described [28]. After permeabilization in 0.5% Triton X-100 for 10 min, any non-specific signal was blocked with 3% BSA for 1 h at RT. The sections were then incubated with primary antibodies FBXO31 (1:100; ab198865; Abcam), DDX4 (1:200; ab27591; Abcam), Foxl2 (1:100; ab5096; Abcam), γ-H2A.X (1:200; ab81299; Abcam), GADD45A (1:100; sc-6850, Santa Cruz Biotech) overnight at 4°C. After PBS washes, they were incubated with corresponding secondary antibodies for 1 h at RT, with DAPI counterstained for 10 min. Finally, the sections were washed and mounted. Fluorescent signals were observed and imaged with a confocal laser scanning microscope (LSM880, Carl Zeiss).

### Annexin V/PI apoptosis assay

Cell apoptosis was detected by fluorescein isothiocyanate (FITC)-labeled Annexin V/propidium iodide (PI) Apoptosis Detection Kit (556547, BD Biosciences, USA) according to the manufacturer’s instructions. Briefly, stably infected KGN cells were cultured in the medium supplemented with either 20 µM Pifithrin-α (PFT-α, Santa Cruz Biotech), 10 mM N-acetyl-l-cysteine, or neither. They were harvested after 6 h incubation, washed with ice-cold PBS, resuspended in 500 μl 1 × binding buffer supplemented with 5 μl Annexin-V-FITC and 1 μl PI for 30 min, and left in the dark at RT. Flow cytometric analysis was performed on a BD FACS Aria Fusion (BD Biosciences). Data acquisition and analysis were performed using FlowJo V8 software (TreeStar, USA).

### EdU assay

The GCs were transfected with FBXO31 siRNA, incubated with 5-ethynyl-20-deoxyuridine (EdU, C10310-1, Ribobio) for 2 h, and processed according to the manufacturer’s instruction. After three washes with PBS, the cells were fixed in 4% PFA for 30 min at RT and then treated with 2 mg/ml glycine. PBS with 0.5% Triton X-100 was used to permeabilize the cells for 10 min. The cells were incubated in 1×Apollo^®^567 reaction cocktail for 30 min after three washes. Then, the DNA contents of the cells in each well were stained with 5 μg/ml Hoechst 33342 for 30 min, and images were captured under a confocal laser scanning microscope.

### Yeast two hybridization (Y2H) assay bait MDM2

For the Y2H assay, the full-length CDS of MDM2 was cloned into pGBKT7 to generate the pGBKT7-MDM2 recombinant plasmid, which was transformed into Y2H Gold yeast to generate bait. A transcription self-activation assay showed that no transcriptional self-activation was found. The same method was used to construct the PGADT7-FBXO31 and PGADT7-F-box-mut recombinant plasmids. Seven groups were designed to test the interactions between MDM2 and FBXO31/F-box-mut: pGBKT7-53&PGADT7-T (positive control), pGBKT7-lam&PGADT7-T (negative control), pGBKT7-MDM2&PGADT7 (to verify MDM2 self-activation), pGBKT7-MDM2&PGADT7-FBXO31 and pGBKT7-MDM2& PGADT7-F-box-mut (to verify interactions). As before, these plasmids were transformed into Y2H Gold. All groups were first cultured on SD/-Leu/-Trp medium (DDO) to determine successful transformation based on the presence of colonies. Further interaction experiments were performed on both SD/-Leu/-Trp/-His medium (TDO) and SD/-Leu/-Trp/-His/-Ade medium (QDO). The verification of possible interactions was determined based on the growth status and blue color of colonies after three days of culture.

### Animals and microinjection of lentivirus into mouse ovaries

Three-week-old Institute of Cancer Research (ICR) mice were purchased from the SPF Biotechnology Co., Ltd (China) and maintained in the Animal Laboratory Center of Institute of Zoology, Chinese Academy of Sciences on a 12/12h light/dark cycle with food and water available ad libitum. All procedures involving mice were approved by the Animal Research Committee and the Ethics Committee of Beijing Obstetrics and Gynecology Hospital, Capital Medical University. After being anesthetized, the mice were placed in a prone position. Then, the back skin and muscles were incised, and bilateral ovaries were carefully removed. In viewing under the microscope, the ovarian membranes were lifted by tweezers to make a space between the membrane and ovary, thus avoiding injury during microinjection. Mice were randomly divided into two groups for microinjection of LV-FBXO31-3xFlag-ZsGreen-puro (FBXO31 group, n=15) or LV-ZsGreen-puro (Vector group, n=15) with 34G needle and lentivirus constructed by Sangon Biotech (China). The dosage of lentivirus particles for one ovary was approximately 6 μl. One week after the lentivirus infection, the mice were sacrificed, and the ovaries were collected immediately for the following analyses.

### Estrous cycle determination

Vaginal smears were obtained at 9:00 am daily for 14 consecutive days using saline solution. Dried smears were fixed with Wright’s Stain solution (G1040; Solarbio) for 3 min, mixed with an equal amount of distilled water, and stained for an additional 5 min. Smears were washed with distilled water and dried at RT. The slides were then examined under a light microscope. The stage of the cycle was determined as follows: proestrus (nucleated epithelial cells mainly), estrus (cornified cells mainly), and metestrus/diestrus (leukocytes in fair abundance).

### Superovulation

Mice were injected intraperitoneally with 5 units of pregnant mare serum gonadotropin (PMSG, Sansheng Inc., China) one week after the lentivirus microinjection. After 48 h, mice were injected with 5 units of human chorionic gonadotropin (hCG; Sansheng Inc.) intraperitoneally to induce ovulation. After 12-14 h, the ovulated oocytes were retrieved from the ampulla of the oviduct and collected for the following experiments.

### ElISA assay

The human estrogen and progesterone levels in the culture supernatants of stably infected GCs were monitored according to the standard ELISA methods (mlbio, China). Results were expressed in nanograms of estrogen/progesterone per microgram protein and then normalized to the vector group. Mouse serum anti-Mullerian hormone (AMH), follicle-stimulating hormone (FSH), estradiol (E_2_), and luteinizing hormone (LH) levels were also monitored by the same methods. According to the manufacturer’s instructions, 96-well plates with antibodies were incubated with supernatants or serum. The indicator concentrations were determined by absorbance at 450 nm using PerkinElmer/Ensight (USA).

### H&E staining, Masson trichrome staining and ovarian follicle counting

Ovaries infected with lentivirus were removed from the mice and fixed in 4% PFA for six hours. The ovaries were then embedded in paraffin and sectioned into 5 μm serial sections. The tissues were stained with Masson trichrome (Masson’s Trichrome Stain Kit, Solarbio) or H&E staining (H&E Stain Kit, Beyotime) according to the manufacturer’s instructions. For ovarian follicle counting, every fifth section was subjected to H&E staining. The number of follicles at different developmental stages was counted blindly in every fifth section throughout the ovary.

### TUNEL assay

The apoptosis assessment was performed by TUNEL BrightRed Apoptosis Detection Kit (Vazyme) following the manufacturer’s instructions. Briefly, ovarian sections were prepared through deparaffinization, rehydration, and proteinase K treatment. Cells climbing to the carrier sheet glass were washed with PBS, fixed with 4% PFA and treated with 0.2% TritonX-100. The sections or cell slides were incubated for 60 min at 37°C in TUNEL reaction solution (5:1 Label Solution: Enzyme Solution). The nuclei were finally stained with DAPI for 20 min at RT. The sections or cell slides were then washed with PBS three times and mounted with an anti-fade mounting medium. Fluorescent signals were observed and imaged with a confocal laser scanning microscope on at least three sections.

### Measurement of reactive oxygen species (ROS) level

Stably infected KGN cells were harvested and resuspended in Opti-MEM (Gibco) containing 10 μM DCFH-DA (Beyotime) for 30 min at 37°C in the dark. The intracellular ROS level was determined by measuring the fluorescence of 2’, 7’-dichlorofluorescein (DCF), the oxidized product of DCF diacetate (DCFH-DA). Cells were then resuspended in PBS and analyzed by BD FACS Aria Fusion. Data acquisition and analysis were performed using FlowJo V8 software.

Oocytes were incubated in Opti-MEM containing 10 μM DCFH-DA for 20 min at 37°C. Following three washes, oocytes were transferred and covered with mineral oil for imaging under LSM880.

### ATP measurement

The ATP content was calculated using the formula derived from the linear regression of standard curve. For each experiment, a minimum of one hundred oocytes were loaded in 1.5 ml tubes and then stored at -80°C until ATP content measurement. When measuring ATP, 200 μl lysis buffer from an ATP detection kit (Beyotime) was added to each tube, and the lysates were centrifuged at 12,000 × g for 5 min at 4°C. The supernatants were transferred to a new 1.5 ml tube and measured using a luminometer (PerkinElmer/Ensight). The relative ATP level was calculated as the following formula: relative ATP level = ATP value/protein value.

### Statistical analysis

For normally distributed data, data were reported as mean ± SD, while Student’s *t*-test was used for parametric testing. Otherwise, log-transformed data were used, and the Mann-Whitney U test was used for nonparametric testing. The correlation between the variables was performed using Pearson rank correlation analysis. For the comparison of multiple groups, one-way ANOVA was used. Significance was evaluated using data from at least three independent experiments, with a *p*-value < 0.05.

## RESULTS

### FBXO31 is upregulated in granulosa cells of POI patients and specifically targeted by miR-106a-5p

**Figure 1.**
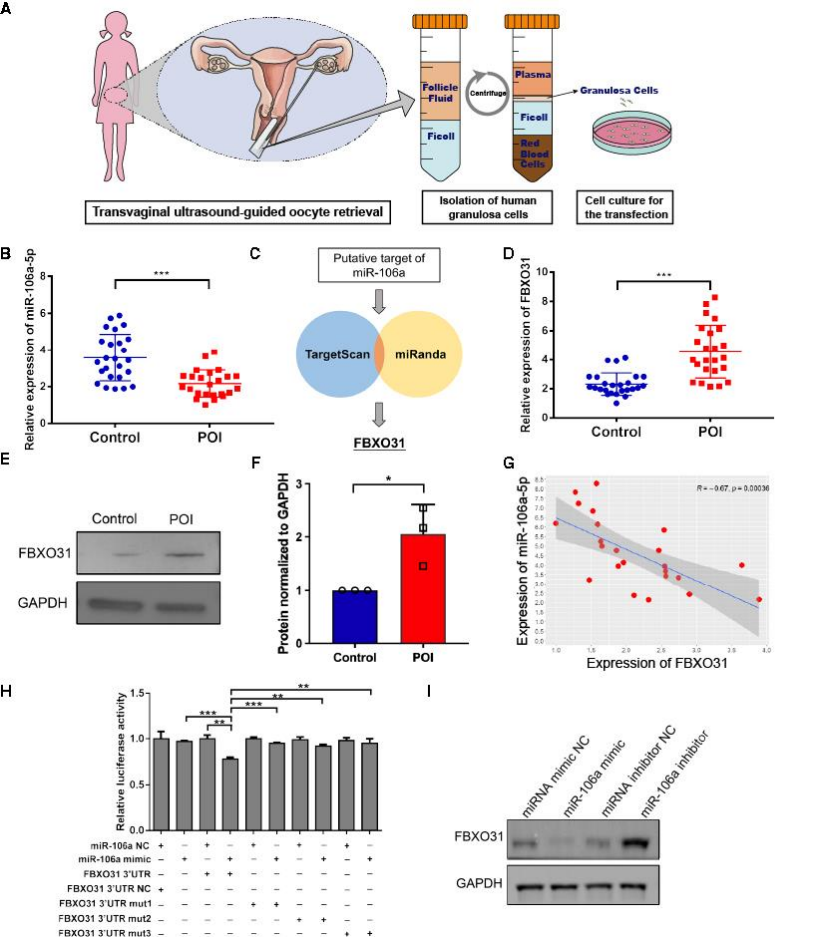
**FBXO31 is upregulated in granulosa cells (GCs) from women with POI and is directly targeted by miR-106a-5p**. **(A)** Schematic procedure of primary hGCs collection. **(B)** The expression level of miR-106a-5p in GCs from control and POI patients (n=49, gene expressions were normalized to U6). **(C)** Diagram showing the approach used to identify putative targets of miR-106a-5p by in silico software prediction. **(D)** The expression level of FBXO31 in GCs from control and POI patients (n=49, gene expressions were normalized to GAPDH). **(E)** WB results showing protein levels of FBXO31 in GCs from control and POI patients. **(F)** Quantified FBXO31 protein levels of Figure 1E (n=3 experiments, data were normalized to GAPDH). **(G)** Correlation of miR-106a-5p and FBXO31 expression in POI patients evaluated by Pearson’s coefficient. (n=49, gene expressions were normalized to U6 and GAPDH respectively). **(H)** Dual-luciferase reporter assay targeting the relationship between FBXO31 and miR-106a-5p. **(I)** WB results showing FBXO31 levels in granulosa cells transfected with miRNA mimic NC, miR-106a mimic, miRNA inhibitor NC, or miR106a-inhibitor (miR-106a-5p, microRNA-106a-5p; NC, negative control). All data were shown as mean ± SD. *p<0.05, ***p<0.001 by Student’s *t*-test (B and D) or Mann-Whitney U test (F).

We previously demonstrated the downregulation of miR-106a in granulosa cells and serum of women with DOR. Here, we collected granulosa cells from 24 POI patients, as well as 25 matched controls undergoing in vitro fertilization-embryo transfer (IVF-ET) (Table 1, Fig. 1A). In line with our earlier research, the expression of miR-106a-5p was significantly decreased among 24 shortlisted POI granulosa cell samples (Fig. 1B) [25]. Then we performed in silico analysis to search for potential targets of miR-106a-5p by using the TargetScan and MiRanda target prediction algorithms, in which FBXO31 was identified (Fig. 1C). Also, FBXO31 mRNA level was significantly increased in granulosa cells from POI patients (Fig. 1D), with a consistent trend of FBXO31 protein levels in WB analysis (Fig. 1E-F). Thereafter, a noticeably negative correlation was observed between the expression of miR-106a-5p and FBXO31 in granulosa cells (Fig. 1G). Considering the regulatory relationships between them, we retrieved the sequence of predicted binding sites in the 3’ untranslated regions (3’UTRs) of FBXO31, which is highly conserved across species (Supplementary Fig. 1A), from the TargetScan database (www.targetscan.org) with predicted scores (Supplementary Fig. 1B). And we performed a dual-luciferase reporter assay to validate these binding sites with three FBXO31 3’UTR-mutants, including base alterations in the nucleotide stretches of 75-81nt, 145-151nt, and double mutants (Supplementary Fig. 1C). Co-transfection of miR-106a mimic and FBXO31 3’UTR mutants resulted in a significantly decreased luciferase activity compared to the other groups, proving the existence of two binding sites (Fig. 1H). To further confirm the negative regulation between FBXO31 and miR-106a-5p, we transfected primary hGCs with miR-106a mimic or inhibitor. We detected that miR-106a mimic significantly downregulated the expression of FBXO31, whereas miR-106a inhibitor increased the level of FBXO31 (Fig. 1I). Taken together, our results confirmed that FBXO31 is upregulated in granulosa cells of POI patients and acts as the direct target gene of miR-106a-5p.

**Table 1:** General Characteristics of patients with primary ovarian insufficiency and unaffected control women.

Characteristics	Control group (n=25)	POI group (n=24)	*P* value
**Age, years**	32.88±3.93	37.50±3.61	0.0001
**BMI, kg/m^2^**	23.65±2.97	22.04±2.51	0.0464
**Basal FSH, IU/L**	7.00±1.09	30.06±5.41	0.0000
**Basal LH, IU/L**	3.39±1.12	9.27±6.50	0.0001
**Basal E_2_, pg/mL**	35.22±11.83	36.13±25.56	0.8728
**Antral follicle number, n**	10.84±3.48	2.29±1.33	0.0000

Values are presented as mean ± standard deviation. Statistical differences were calculated using the non-parametric Kruskal-Wallis H test. Abbreviations: BMI, body mass index; FSH, follicle-stimulating hormone; LH, luteinizing hormone; E_2_, estradiol

### FBXO31 elevates apoptosis of granulosa cells

**Figure 2.**
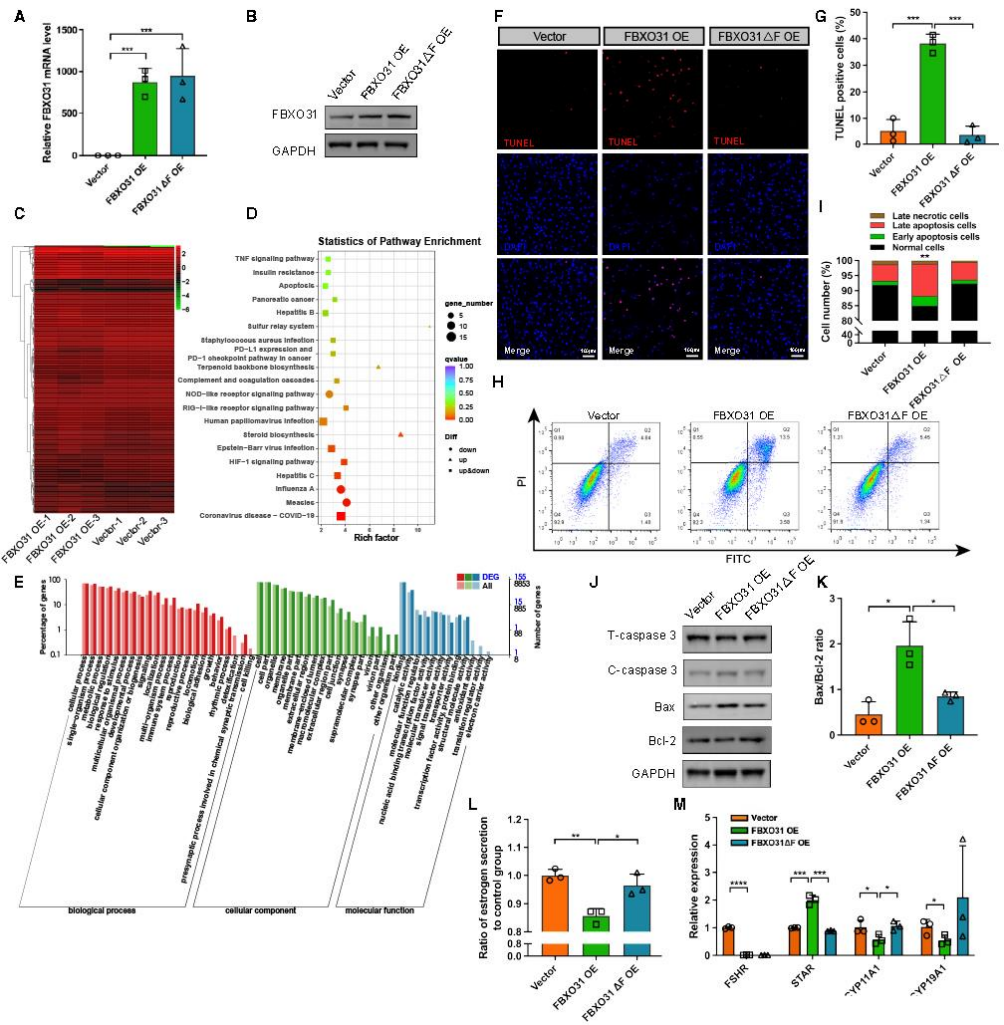
**FBXO31 overexpression induces apoptosis of granulosa cells**. **(A)** The expression level of FBXO31 in KGN cells transfected with Vector, FBXO31 OE and FBXO31△F OE lentivirus. The lentivirus with an empty vector (Vector) was used as a control. (n=3 per group, gene expressions were normalized to GAPDH) (B) WB results showing increased levels of FBXO31 in KGN cells transfected with FBXO31 OE and FBXO31△F OE lentivirus. **(C)** Clustered heatmap showing differentially expressed genes (DEGs) in FBXO31-overexpressed cells compared with the vector group. (n=3 per group). **(D)** Bulb map of KEGG analyses of DEGs in FBXO31-overexpressed cells compared with the vector group. **(E)** Bar plot of highly enriched GO terms between the FBXO31 OE and vector group. The *x*-axis displays GO terms. The left *y*-axis represents the percentage of genes, and the right *y*-axis represents the number of genes. The bar color corresponds to different GO categories, with red for biological processes, green for cellular components, and blue for molecular functions. **(F)** Representative images of apoptotic cells reflected by TUNEL staining in indicated groups. Scale bars: 100 μm. **(G)** Statistics of TUNEL-positive cells quantified by counting the cells with fluorescent signal (n=3 per group). **(H)** Apoptotic cells were monitored by Annexin-V-FITC/PI staining and flow cytometry analysis. The lower-right quadrant of each plot shows early apoptotic cells, whereas the upper-right quadrant shows late apoptotic cells. **(I)** Percentages of Annexin V-FITC/PI-positive cells from gated cells. Each experiment was performed in triplicate, and similar results were obtained each time. **(J)** WB results showing the effects of overexpressing FBXO31 on the levels of apoptosis-related proteins, including caspase-3, Bax, and Bcl-2. **(K)** Apoptotic index Bax/Bcl-2 was measured in Figure 2J (n=3 per group, data were normalized to GAPDH). **(L)** Estrogen levels in the culture supernatants of FBXO31-overexpressed cells (n=3 per group, results were represented in nanograms of estrogen per microgram protein and then normalized to the vector group). **(M)** The expression profiles FSHR and steroidogenic enzymes in FBXO31-overexpressed cells (n=3 per group, gene expressions were normalized to GAPDH). All data were shown as mean ± SD. *p<0.05, **p<0.01, ***p<0.001, ****p<0.0001 by Mann-Whitney U test (A, G, K, L, M) or Chi-square test (I).

To gain insights into how FBXO31 overexpression affects granulosa cells, we have established the FBXO31-overexpressed (OE) KGN cells (immortalized human granulosa cells) with lentivirus (Fig. 2A-B, Supplementary Fig. 2A-C). We first analyzed the transcriptome of FBXO31 OE cells that enriched with GFP-positive in FACS and screened 200 upregulated genes and 177 downregulated genes with a |fold change (FC)| ≥ 1.5 and false discovery rate (FDR) < 0.01 (Fig. 2C, Supplementary data). We confirmed the expression pattern of two upregulated genes and two downregulated genes identified in the RNA-seq by qRT-PCR (Supplementary Fig. 2D-E). In FBXO31 OE cells, steroid biosynthesis and apoptosis were listed in the top 20 pathways of KEGG analysis, as they were mainly associated with the functions of ovarian GCs (Fig. 2D). Meanwhile, terms including “reproduction”, “reproductive process” and “growth” were among the most enriched biological processes according to the GO analysis (Fig. 2E).

Thus, we examined the cell apoptosis level of granulosa cells overexpressing FBXO31 to validate the possible roles of FBXO31 in the ovary. Since the F-box domain is essential for FBXO31 in ubiquitination, we also constructed a mutant FBXO31 (FBXO31ΔF) lentivirus with the F-box domain deleted (Fig. 2A-B). In the FBXO31 OE group, a higher ratio of TUNEL (TdT-mediated dUTP Nick-End Labeling)-positive cells was observed (Fig. 2F-G). To measure the extent of apoptosis in FBXO31 OE cells, we performed a flow cytometry analysis using Annexin-V-FITC/PI labeling and identified a significant increase of apoptotic cells in the FBXO31 OE group (13.5%) compared to the vector and FBXO31ΔF groups (4.84% and 5.45%) (Fig. 2H-I). When evaluating the expression of common apoptosis-related proteins, WB results demonstrated that FBXO31 overexpression increased the level of cleaved caspase-3 (C-caspase-3) and B-cell lymphoma 2 associated X protein (Bax), while reduced B-cell lymphoma 2 (Bcl-2) in GCs (Fig. 2J).

Considering the apoptotic index Bax/Bcl-2 was significantly increased in the FBXO31 OE cells, but not in the FBXO31△F OE cells (Fig. 2K), FBXO31 was established to induce cell apoptosis relying on the F-box domain. Additionally, this viewpoint was supposed by the expression patterns of apoptosis-related genes identified in the RNA-seq (Supplementary Fig. 2F-G). Cathepsin S (CTSS), serving as a member of the cysteine protease family and participating in the regulation of rabbit granulosa cell proliferation via the upregulation of Bcl-2, was decreased in FBXO31 OE cells. DNA Damage Inducible Transcript 3 (DDIT3) and Growth Arrest and DNA Damage Inducible Alpha (GADD45A), being reported to regulate apoptosis effectors and participate in other degenerative processes, were both elevated in FBXO31 OE cells. Furthermore, other functional alterations of FBXO31 presented on granulosa cells were found. EdU (5-Ethynyl-2’-deoxyuridine) staining was performed to observe the cell proliferation in FBXO31 knockdown (KD) cells (Supplementary Fig. 2H), in which the proliferation rate is elevated markedly in FBXO31 KD groups (Supplementary Fig. 2I). The estrogen secretion in the supernatants of FBXO31 OE cells decreased significantly (Fig. 2L), which may be account by the lower expression of FSHR, CYP11A1 and CYP19A1 (Fig. 2M), while the progesterone secretion represented no difference (Supplementary Fig. 2J-K). Taken together, our data indicated that FBXO31 overexpression impaired the functions of granulosa cells, especially inducing the apoptosis of granulosa cells.

**Figure 3.**
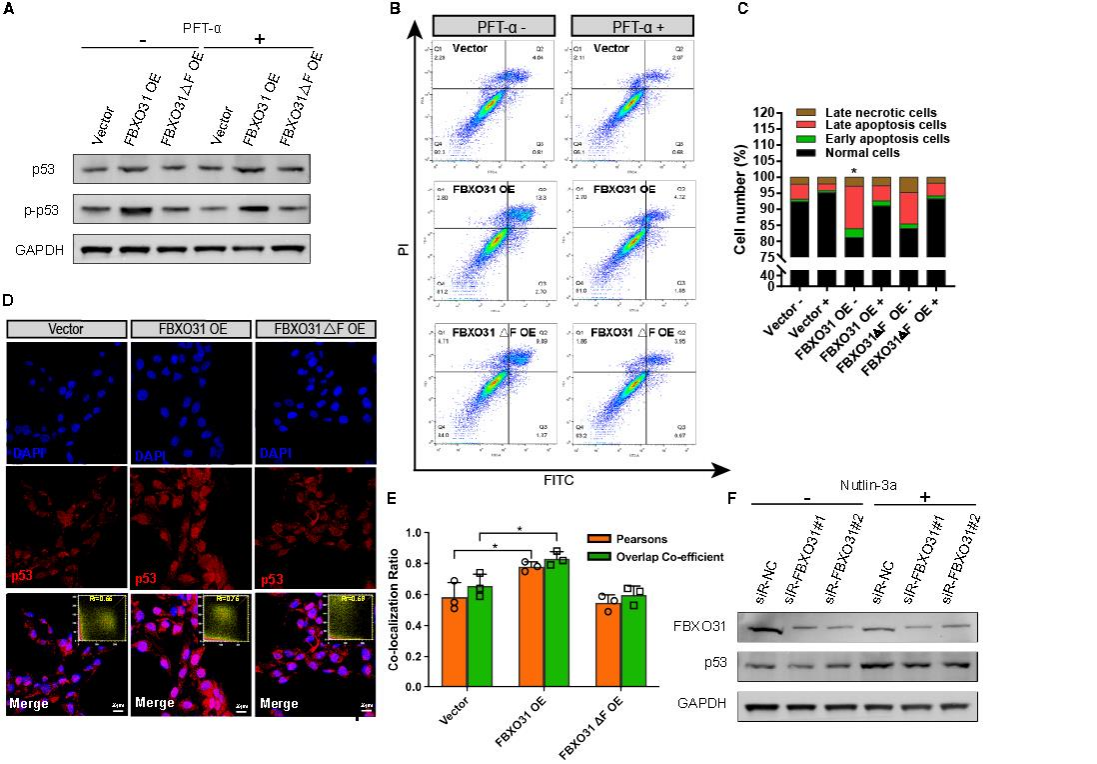
**Activation and translocation of p53 in FBXO31 OE cells induce apoptosis**. **(A)** WB results showing p53 and p-p53 levels in FBXO31 OE and FBXO31△F OE cells with or without PFT-α treatment. **(B)** Apoptotic cells were monitored by Annexin-V-FITC/PI staining and flow cytometry analysis. The lower-right quadrant of each plot shows early apoptotic cells, whereas the upper-right quadrant shows late apoptotic cells. **(C)** Percentages of Annexin V-FITC/PI-positive cells from gated cells. -, treatment without PFT-α; +, treatment with PFT-α. Each experiment was performed in triplicate and similar results were obtained each time. (*p<0.05 by Chi-square test). **(D)** Immunofluorescence assays showing the intracellular distribution of p53 in FBXO31 OE and FBXO31△F OE cells. Scale bars: 20 μm. **(E)** Quantitative co-localization analysis of p53 and nuclei of the cells shown in panel Merge of Figure 3D by using the Colocalization Finder Plugin of ImageJ software. (n=3, *p<0.05 by Pearson’s correlation and overlap coefficient). **(F)** WB results showing p53 levels in FBXO31 KD granulosa cells treated with or without nutlin-3a. -, treatment without nutlin-3a; +, treatment with nutlin-3a.

### FBXO31 induces apoptosis through p53 signaling in granulosa cells

Tumor suppressor p53 plays a significant role in apoptosis and growth arrest. When under stress, p53 accumulates rapidly, activates downstream biological processes, and induces cell growth arrest and apoptosis [29, 30]. As a prominently affected downstream effector, p53 is believed to contribute to the cell apoptosis induced by FBXO31 overexpression. In this, we first examined the expression levels of p53 and p-p53 (the activated form of p53) in the granulosa cells treated with the Pifithrin-α (PFT-α, a p53 inhibitor) after the lentivirus transfection. PFT-α hardly suppressed the increase of p53 level induced by FBXO31 overexpression in granulosa cells, however, it mildly inhibited the activated p-p53 (Fig. 3A). Next, we investigated whether the inhibition of p-p53 alleviated the apoptosis in FBXO31 OE cells. By using flow cytometry analysis, we monitored a lower percentage of apoptotic cells in the FBXO31 OE group after the treatment of PFT-α as expected (Fig. 3B-C), reinforcing the finding that cell apoptosis in the FBXO31 OE group is implemented by activating p53. Additionally, with the employment of immunofluorescence assay in FBXO31 OE cells, we observed a markedly higher expression of p53, along with an increased co-localization with the nucleus (Fig. 3D). Coinciding with a prior study [31], with the Pearson coefficient of DAPI and p53 was as high as 0.75, the enhanced p53 nuclei accumulation could lead to p53 activation (Fig. 3D-E). As p53 can be degraded through MDM2/p53 axis [21, 32], thereafter, nutlin-3a, which activates p53 by disrupting MDM2-p53 interaction and induces a p53-mediated cell cycle arrest and apoptosis [32], was used in our study. As shown in Fig. 3F, nutlin-3a increased p53 expression in all groups and alleviated reduced p53 expression in FBXO31 KD granulosa cells. Overall, these results showed that the activation and nuclear translocation of p53 play pivotal roles in the granulosa cell apoptosis triggered by FBXO31 overexpression, especially through MDM2/p53 axis.

### FBXO31 governs p53 by degrading MDM2 in human granulosa cells

**Figure 4.**
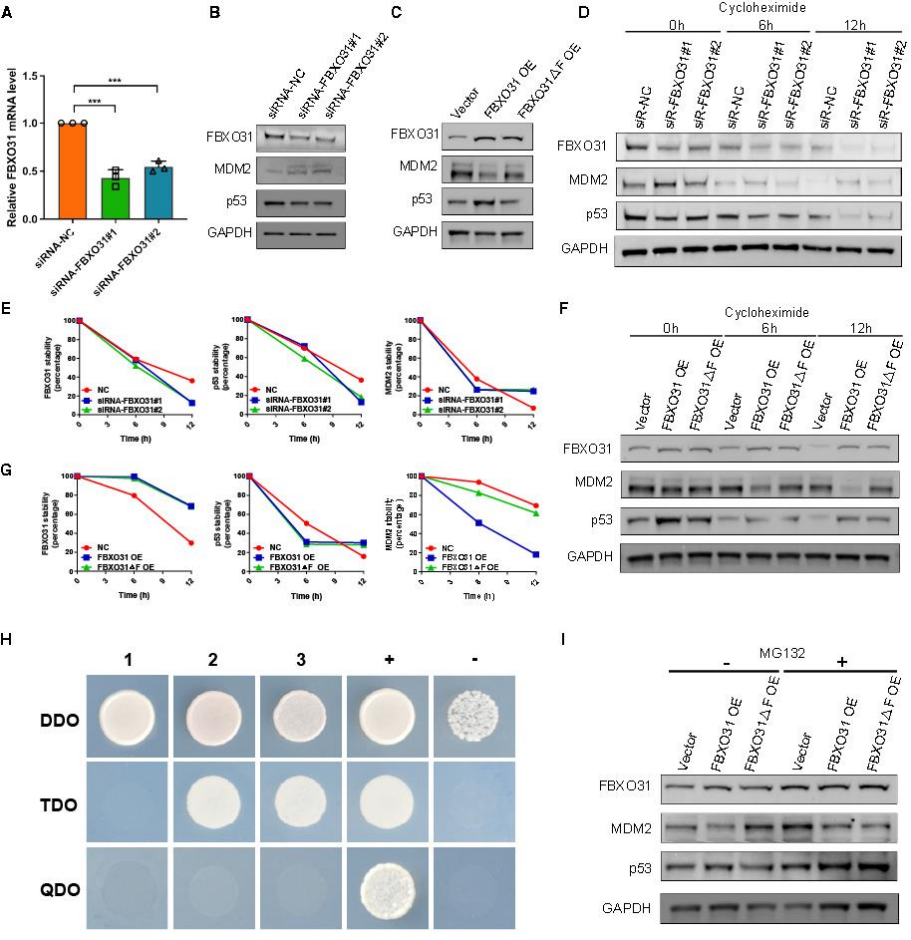
**P53 regulates granulosa cell apoptosis via FBXO31-mediated loss of MDM2**. **(A)** The expression level of FBXO31 in KGN cells transfected with siRNA-targeted FBXO31 (n=3 per group, gene expressions were normalized to GAPDH, ***p<0.001 by Mann-Whitney U test). **(B)** WB results showing decreased expression of p53, as well as increased MDM2 levels in FBXO31 KD granulosa cells. **(C)** WB results showing an increased p53 level, with a decreased MDM2 level in FBXO31-overexpressed granulosa cells. **(D)** Immunoblot assay of MDM2 stability in FBXO31 KD and FBXO31△F KD cells following treatment with cycloheximide. **(E)** Quantification of the cycloheximide-chase assay in Figure 4D, showing the ratio of relative levels of MDM2. Time 0 was set to 100%. (Data were normalized to GAPDH). **(F)** Immunoblot assay of MDM2 stability in FBXO31 OE and FBXO31△F OE cells following treatment with cycloheximide. **(G)** Quantification of the cycloheximide-chase assay in Figure 4F. (Data were normalized to GAPDH). **(H)** Y2H assay showing that FBXO31 (prey) interacts with MDM2 (bait). DDO, SD/-Leu/-Trp medium; TDO, SD/-Leu/-Trp/-His medium; QDO, SD/-Leu/-Trp/-His/-Ade medium. 1, pGBKT7-MDM2&PGADT7; 2, pGBKT7-MDM2&PGADT7-FBXO31; 3, pGBKT7-MDM2&PGADT7-F-box-mut; +, pGBKT7-53&PGADT7-T; -, pGBKT7-lam&PGADT7-T. **(I)** Immunoblot monitoring MDM2 and p53 levels in FBXO31 OE and FBXO31△F OE cells in the presence or absence of MG132. The data shown are the representative images of three independent experiments with similar results.

It has been reported that loss of MDM2 mediated by FBXO31 leads to elevated p53, resulting in growth arrest following genotoxic stress [21]. To verify whether a similar effect exists in ovarian granulosa cells, we performed an RNAi experiment to confirm the regulation of p53 by endogenous FBXO31. After validating a significant decrease of FBXO31 in granulosa cells transfected with siRNA- FBXO31#1 or #2 (Fig. 4A), p53 was decreased with the increase of MDM2 levels (Fig. 4B). When FBXO31 was elevated by lentivirus, ectopic expression of FBXO31ΔF failed to decrease the MDM2 level or increase the p53 level in contrast to wild-type FBXO31 (Fig. 4C). Next, to verify the function of FBXO31 in regulating the stability of proteins, we measured the half-lives of MDM2 and p53 with a cycloheximide-chase assay in FBXO31 KD cells. Here, the half-life of MDM2 was substantially longer in FBXO31-downregulated cells (Fig. 4D-E). The p53 stability, in agreement with previous studies [21, 22], appeared to be in an opposing trend with MDM2 (Fig. 4D-E). When we monitored the half-lives of MDM2 and p53 after overexpressing FBXO31 and FBXO31ΔF, the ectopic FBXO31 (not FBXO31ΔF) substantially reduced the half-life of MDM2 in granulosa cells. But in both FBXO31 OE and FBXO31ΔF OE groups, the half-life of p53 persisted after 12-hour cycloheximide treatment (Fig. 4F-G). We subsequently tested the direct interaction between FBXO31 and MDM2 proteins by using a yeast two hybridization (Y2H) system and revealed that both FBXO31 and F-box domain mutant interact intensively with MDM2 (Fig. 4H). To validate whether MDM2 is regulated by FBXO31 via a proteasome-mediated protein degradation process, we furtherly measured the protein levels of FBXO31 OE cells treated with or without the proteasome inhibitor MG132, which minimizes the degradation of polyubiquitinated proteins. The results showed that the MDM2 degradation mediated by FBXO31 was blocked by the addition of MG132 (Fig. 4I). Altogether, our results indicated that FBXO31 facilitates p53 expression through direct interaction with MDM2 in human granulosa cells.

### FBXO31 triggers ROS accumulation and induces mitochondrial damage through the Bax/Bcl-2 pathway

**Figure 5.**
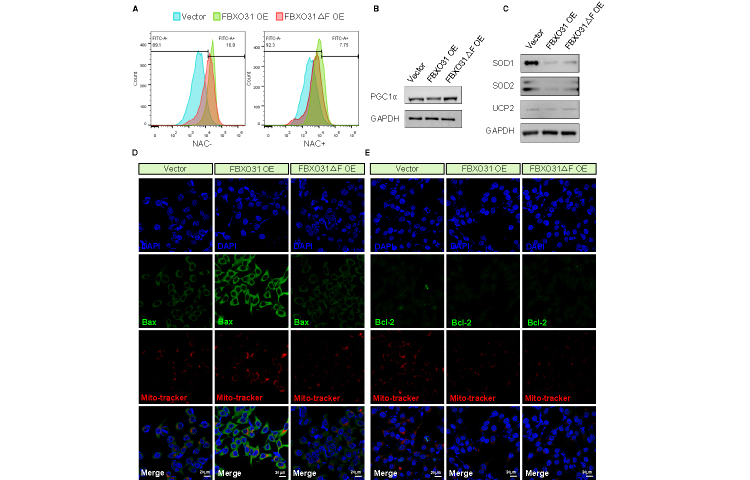
**FBXO31 triggers ROS accumulation and alters the subcellular expression of Bax/Bcl-2**. **(A)** ROS levels detected by fluorescence intensity of DCF using flow cytometry. Data were analyzed using FlowJo software. **(B)** WB results showing PGC1α level in FBXO31 OE and FBXO31△F OE cells. **(C)** WB results showing the antioxidant enzymes SOD1, SOD2, and UCP2 levels in FBXO31 OE and FBXO31△F OE cells. (D, E) Representative images of the Bax (D) and Bcl-2 (E) in FBXO31 OE cells, with mitochondria stained with mitotracker. Scale bars: 20 μm. The data shown are the representative images of three independent experiments with similar results.

Apoptosis is generally regulated by the intrinsic mitochondrial mechanisms and the activation of pro-apoptotic p53/Bcl-2 family proteins (acting on the mitochondrial outer membrane permeabilization) [33]. Therefore, we measured the cellular ROS level, which may get involved in mitochondrial damage. The results revealed that cells overexpressing FBXO31 generated more ROS, while NAC (a specific inhibitor of ROS) treatment significantly decreased the ROS production (Fig. 5A). By this way, NAC lessen the apoptosis brought by FBXO31 overexpression, as a decreased ratio of apoptotic cells captured in our flow cytometry analysis and TUNEL staining (Supplementary Fig. 3A-C). Collectively, our results suggested that the overexpression of FBXO31 causes GC apoptosis by triggering ROS accumulation. Additionally, a recent study showed that p53 can bind and repress the promoter of peroxisome proliferator-activated receptor-gamma (PPARγ) coactivator-1α (PGC1α), a master regulator of mitochondrial biogenesis and function [34]. We measured the expression levels of PGC1α to ascertain if this mitochondrial pathway is also implicated. Expectedly, PGC1α showed a lower expression in the FBXO31 OE group compared to the other groups (Fig. 5B).

Given that PGC1α is a crucial component in the induction of several antioxidants, we detected the expression of multiple antioxidant enzymes in FBXO31 OE cells, including superoxide dismutase 1 (SOD1) and superoxide dismutase 2 (SOD2), and uncoupling protein 2 (UCP2). As shown in Figure 5C, FBXO31-mediated PGC1α deficiency also lowered the antioxidant defenses of cells against the rise of ROS. Next, we observed an obvious increase of Bax and a decrease of Bcl-2 expression in FBXO31 OE cells by immunofluorescence (Fig. 5D). As an anti-apoptotic factor, Bcl-2 could bind and interact with Bax to prevent the formation of mitochondrial pores and thus inhibit the execution of apoptosis [35]. However, the increased translocation of Bax to mitochondria (identified by the enhanced Bax green fluorescence overlaying with mitochondrial red fluorescence) in FBXO31 OE cells might involve in the excessive ROS generation, and lower Bcl-2 expression (Fig. 5E). Furthermore, NAC treatment restored Bcl-2 and alleviated the Bax translocation (Supplementary Fig. 3D-F). From the above observations, we suggested that excessive ROS is relevant to mitochondrial damage through the regulation of Bax/Bcl-2 expression in FBXO31 OE cells.

**Figure 6.**
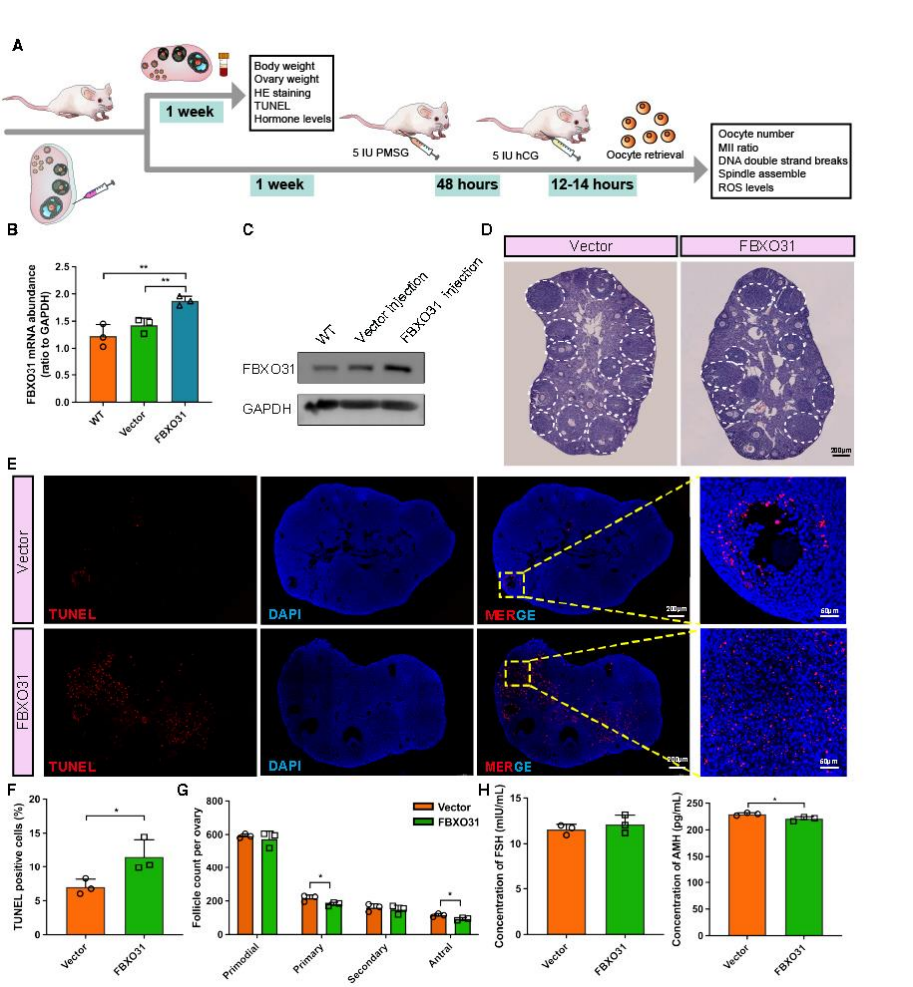
**FBXO31 overexpression leads to follicular atresia in mouse ovaries**. **(A)** Schematic procedure of lentivirus microinjection to mouse ovaries and experimental indexes. **(B)** FBXO31 mRNA levels in the mouse ovaries microinjected with lentivirus. (n=3 per group, GAPDH was used to normalize the results). **(C)** WB analysis of FBXO31 levels in the mouse ovaries microinjected with lentivirus (n=3 per group). **(D)** Representative H&E staining images of mouse ovaries in the FBXO31 and vector group. Large antral follicles are denoted with white broken lines. Scale bars: 200 µm (n=3 per group). **(E)** TUNEL staining showing the atretic follicles of ovarian sections from indicated groups. Apoptotic cells, red; cell nuclei, blue. Scale bars: 200 μm for the left images. Scale bars:50 μm for the rightmost image. **(F)** Quantification of TUNEL-positive cells by counting the cells with the fluorescent signal using the software ImageJ (n=3 per group). **(G)** Quantification of follicles in each developmental stage (primordial, primary, secondary, and antral) (n=3 per group). **(H)** ELISA results showing effects of FBXO31 OE lentivirus microinjection on serum FSH and AMH levels. All data were reported as mean ± SD. *p<0.05, **p<0.01 by Mann-Whitney U test (B, G, H) or Chi-square test (F).

**Figure 7.**
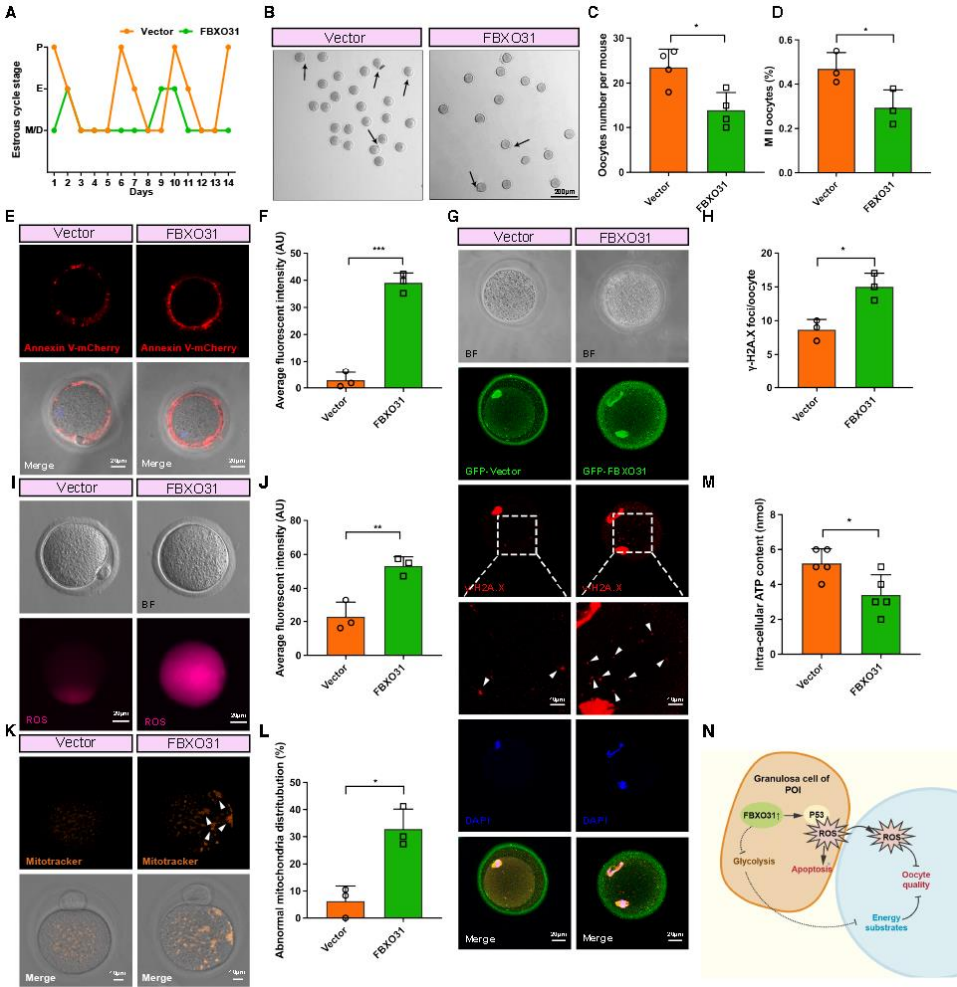
**FBXO31 overexpression impairs the oocyte quality in mouse ovaries**. **(A)** Representative graphs of irregular estrous cycle in FBXO31 group mice and regular estrous cycle in vector group. (n=5 per group). **(B)** Representative images of oocytes retrieved from the FBXO31 and vector group. Black arrows indicate MII oocytes. Scale bars: 200 μm. **(C)** Numbers of retrieved oocytes per ovary in two groups (n=4 per group). **(D)** Percentage of MII oocytes between two groups (n=3 per group). **(E)** The representative image of the Annexin V signal in the cell membrane and transparent zone of oocytes from two groups. Annexin V-mCherry, Red. Scale bars: 20 μm. **(F)** Quantification of fluorescence intensity of Annexin-V in the vector and FBXO31 groups (n=3 per group). **(G)** Immunofluorescence staining of γ-H2A.X in oocytes retrieved from FBXO31 and vector groups. Nuclei were stained with DAPI. White arrowheads indicated γ-H2A.X foci (n=3 per group). Vector/FBXO31-GFP, green; γ-H2A.X, red; cell nuclei, blue. Scale bars: 20 μm. **(H)** Number of γ-H2A.X foci in oocytes from two groups. (n=3 per group). **(I)** Images of bright fields and ROS levels detected by fluorescence intensity of DCF in oocytes from two groups. ROS, purple. scale bars: 20 μm. **(J)** Quantification of fluorescence intensity of ROS in oocytes from two groups (n=3 per group). **(K)** The typical images of mitochondrial distribution. Mitochondria were stained with mitotracker. White arrowheads indicated the abnormal distribution of clustered mitochondria. **(L)** Quantification of abnormal oocytes with clustered mitochondria from the two groups (n=3 per group). **(M)** The adenosine triphosphate (ATP) contents of mouse oocytes from two groups. (n=5 per group, with a minimum of 100 oocytes in each group). **(N)** The mechanisms diagram of aberrantly high FBXO31 impairs oocyte quality in premature ovarian insufficiency. All data were shown as mean ± SD. *p<0.05, **p<0.01, ***p<0.001 by Mann-Whitney U test (C, F, H, J and M) or Chi-square test (D and L).

### FBXO31 induces follicular atresia in mouse ovaries

After investigating the regulatory mechanisms of FBXO31 in granulosa cells, then we explored the way it alters the follicular microenvironment. In this, mouse ovaries were harvested one week after being microinjected with the GFP-expressed lentiviruses LV-FBXO31-3xFlag-ZsGreen-puro (FBXO31 group) or LV-ZsGreen-puro (Vector group) (Fig. 6A, Supplementary Fig. 4A). By using qRT-PCR and WB, we identified higher FBXO31 mRNA and protein levels in the ovaries transfected with the FBXO31 lentivirus (Fig. 6B-C). Then with the use of immunofluorescence staining, we affirmed the expression of FBXO31 in both cortex and medulla, while GFP indicated no difference between the vector and FBXO31 group (Supplementary Fig. 4B). In the subsequent tests of ovarian and physical development in FBXO31 group, no differences were found in ovarian weight and body weight (Supplementary Fig. 4C-D). Next, we detected the morphological changes of follicles in ovaries successfully overexpressing FBXO31. Hematoxylin and eosin (H&E) staining revealed that fewer large antral follicles resided in the FBXO31 group (Fig. 6D). Given that apoptosis participates in the elimination process of the follicles that are not progressed to ovulation [36], we assessed the follicular apoptosis state with TUNEL assay. In FBXO31 group, we observed an increased proportion of apoptotic granulosa cells among the large growing follicles, which was accompanied by a greater number of atretic follicles (Fig. 6E-F), but not conspicuously in primordial follicles (Supplementary Fig. 4E). Additionally, follicles at different developmental stages were counted in every 5th serial section. FBXO31 overexpression caused a decreased number of follicles at each stage, especially a striking decrease in primary and antral follicles (Fig. 6G). Moreover, we estimated ovarian fibrosis via Masson trichrome staining and found no significant alteration between the two groups, though there seemed to be more collagen fibers in the FBXO31 group (Supplementary Fig. 4F). We also explored the ovarian function by measuring specific serum hormone indicators. Except for a significant decrease of anti-Mullerian hormone (AMH) level in the FBXO31 group, other hormones including FSH (follicle-stimulating hormone), E_2_ (estradiol), and LH (luteinizing hormone) showed no differences between two groups (Fig. 6H, Supplementary Fig. 5A). At last, increased GADD45A and γ-H2A.X expression levels were observed in the growing follicles of FBXO31 group, confirming that the aberrant upregulation of FBXO31 induces damage to the granulosa cells (Supplementary Fig. 5B-C). With the occurrence of DNA damage signals in oocytes, our results suggested that FBXO31 overexpression might impair the oocyte quality as well (Supplementary Fig. 5C). Altogether, our *in vivo* experiment indicated that overexpression of FBXO31 impairs the normal growth of granulosa cells and leads to follicular atresia.

### Aberrantly high FBXO31 in mouse ovary impairs oocyte quality

In order to examine whether FBXO31 overexpression in mouse ovaries causes impaired ovulation, the stages of the estrous cycle were detected in the FBXO31 OE group and the vector group respectively. Regular estrous cycles were found in the vector group, while the FBXO31 OE group displayed irregular estrous cycles (Fig. 7A). To distinguish the significant alterations in ovulation and the quality of oocytes caused by FBXO31 overexpression, we implemented superovulation one week after lentivirus microinjection. Remarkably, the FBXO31 OE group yielded fewer oocytes per ovary (14.00±3.92 oocytes) than the vector group (23.50±4.04 oocytes) (Fig. 7B-C). Moreover, the rate of MII (Metaphase II) oocytes was significantly decreased in the FBXO31 OE group (Fig. 7D). Thereafter, the spindle morphology and chromosome alignment were monitored by immunofluorescence staining of α-tubulin. No spindle abnormality was identified between two groups, and the chromosome alignment appeared overall normal (Supplementary Fig. 6A). However, Annexin V staining showed that oocytes in the FBXO31 OE group exhibited significant positive fluorescence signals, indicating the occurrence of early apoptosis (Fig. 7E-F). As DNA damage is a major trigger of cell apoptosis [37], we measured DNA double-strand breaks (DSBs) in oocytes by γ-H2A.X immunostaining. The ratio of γ-H2A.X foci/oocyte was significantly higher in the FBXO31 OE group compared with the vector group (Fig. 7G-H). As FBXO31 has elevated ROS levels in granulosa cells in our study, we tested the ROS level of the oocytes as well. Remarkably, ROS overproduction was observed in the oocytes overexpressing FBXO31 (Fig. 7I-J). Then we investigated the mitochondrial distribution of MII oocytes in light of its crucial function in ROS production. In the vector group, mitochondria were generally distributed throughout the cytoplasm, while we found more aggregated and clustered mitochondria occurred in the oocytes of the FBXO31 OE group (Fig. 7K). As the abnormal distribution of mitochondria and excessive ROS may lead to a disturbance of energy metabolism, we collected more oocytes for ATP content analysis. The ATP level was clearly lowered in the oocytes of FBXO31 OE group (Fig. 7M). And the decreased energy production of the oocytes may also be related to the decreased supply of energy substrates in granulosa cells, as we discovered that the expression of key glycolytic enzymes ENO1 and ENO2 were significantly decreased in FBXO31 OE granulosa cells (Supplementary Fig. 6B-C). Above all, we found that the overexpression of FBXO31 not only impaired the normal growth of granulosa cells, but also reduced the quantity and quality of oocytes via an excessive accumulation of DNA damage mediated by ROS (Fig. 7N).

## DISCUSSION

POI commonly manifests in hormone imbalances, menstrual disorders (amenorrhea or oligomenorrhea), anovulation, and infertility [38]. The etiology of POI is clinically challenging, and includes genetic, autoimmune, metabolic, and infectious factors [39]. Follicular atresia essentially results from the apoptosis of granulosa cells, and is considered to be the main reason for eliminating follicles in the physiological and pathological processes [40]. Therefore, it is necessary to understand the mechanisms of granulosa cell apoptosis in order to elucidate the pathophysiology of POI. However, the specific regulatory mechanisms concerning the biological processes of ovaries are still poorly understood. In our previous study, downregulated miR-106a was found to contribute to the pathogenesis of DOR by reducing granulosa cell viability and promoting apoptosis via enhanced ASK1 signaling [25]. However, FBXO31, one of the predicted targets of miR-106a-5p, has not yet been investigated in the pathology of ovarian disorders. As our research clue, we identified the decrease of miR-106a-5p in GC samples of patients with POI, with FBXO31 validated as a target. And we further explored the biological role of FBXO31 in granulosa cells. The aberrant upregulation of FBXO31 in granulosa cells of POI women induced the follicular atresia via the p53/ROS signaling pathway, leading to the impairment of ovarian function. However, the link between FBXO31 and miR-106a-5p could be strengthened further.

Serving as the substrate-recognition component of the SKP/Cullin/F-box protein class of E3 ubiquitin ligases, FBXO31 has been previously shown to directly degrade cyclin D1 [18], SNAIL [19], MDM2 [21], and mitogen-activated protein kinase 6 (MKK6) [41], all of which are related to tumorigenesis. Here, we have demonstrated that overexpression of FBXO31 (which is negatively regulated by miR-106a-5p) in the granulosa cells harvested from POI women elevated p53 levels through directly degrading MDM2. It was previously reported that Bcl-2 can bind and interact with Bax to prevent mitochondrial pore formation, which subsequently inhibits the execution of cell apoptosis [35]. We showed here that in the cells overexpressing FBXO31, the expression levels of the apoptotic protein Bax and caspase-3 were higher, while Bcl-2 was in the opposite trend in our WB results. Such induced apoptosis could be prevented by suppressing the binding of MDM2 and p53 with the addition of nutlin-3a. This exactly indicated that MDM2/p53 acts as a critical part in terms of triggering apoptosis in FBXO31 OE granulosa cells. Furthermore, in many cell types, activated p53 is usually associated with DNA damage resulting from UV and γ-radiation, as well as etoposide [42, 43]. Here, the application of PFT-α evidently decreased the apoptosis of FBXO31 OE cells, and elevated p53. This, in turn, participated in the translocation of active Bax to mitochondria, where it has been reported to mediate mitochondrial apoptosis through the transcription-independent pathway [44]. Because this mitochondrial dysfunction is accompanied by the accumulation of ROS (toxic products in cellular metabolism during oxidative stress), we then investigated the change in ROS level after upregulating FBXO31. P53 was able to mediate the repression of PGC1α, thereby inhibiting mitochondrial biogenesis and function, leading to excess ROS [31, 45]. Consistently, we found that ROS was precisely augmented in FBXO31 OE cells, together with the relatively lower expression of a series of antioxidant enzymes. Aberrant PGC1α caused by overexpressing FBXO31 failed to induce the antioxidant enzymes, which may be resulted from the p53-mediated mitochondrial dysfunction. And the inhibition of ROS partly alleviated the apoptosis induced by FBXO31 OE. Collectively, we demonstrated through a series of *in vitro* experiments, that FBXO31, being regarded as an important regulator of p53/ROS pathways, could promote apoptosis of granulosa cells in the occurrence of POI.

It has been well established that granulosa cell apoptosis leads to follicular atresia. Therefore, we looked into the morphology of mouse ovaries after successfully overexpressing FBXO31. FBXO31 OE mice displayed significantly more atretic follicles than the vector group. Additionally, we found a decreased number of oocytes, as well as a lower MII oocyte rate in these mice after superovulation. Previous studies illustrated that excessive ROS production and reduced antioxidant activity are generally existed in aging oocytes, contributing to the age-related decline of competent oocyte development [46]. Here, we assessed the ROS levels of oocytes, as it may exert rather deleterious effects on oocyte quality. Indeed, we observed that FBXO31 overexpression increases ROS levels in oocytes, which is similar to that in the granulosa cells. Growing evidence has demonstrated that the elevated ROS level impairs oocyte quality by disrupting spindle assembly and subsequent chromatin misalignment [47, 48], which we presumably regarded as a reason for our results. However, different from obvious DNA damage, the developing spindle appeared normal in the FBXO31 OE oocytes, suggesting that FBXO31 may not have a substantial impact in our study. All of these clues indicated that FBXO31 may play a role in aggravating follicular atresia and impairing oocytes by breaking double-stranded DNA. In another aspect, oocyte quality is highly dependent on the metabolic support provided by granulosa cells. As the key enzymes of glycolysis, ENO2 was significantly decreased in the FBXO31-overexpressed granulosa cells, accompanied by a slight down-regulation of ENO1. Furthermore, the impaired glycolysis in FBXO31-overexpressed granulosa cells is closely connected with the reduced ATP production in oocytes from the FBXO31 lentivirus group and appeared to be closely related to poor oocyte developmental competence in our study. We will deepen the exploration of the effect of aberrantly high FBXO31 posed on glycolysis, as well as provide more animal evidence to show the impairments of the oocyte quality in the future.

In conclusion, our study strongly indicated that the upregulated FBXO31 expression in granulosa cells promotes apoptosis and follicular atresia via the p53/ROS signaling pathway, which might help explain the occurrence of POI. Hence, we suggested that FBXO31 may serve as a novel marker of POI and provide a new perspective on the potential mechanisms behind POI pathogenesis.

## Supplementary Materials

The Supplementary data can be found online at: www.aginganddisease.org/EN/10.14336/AD.2023.0809.


