# Erratum to “Aberrantly High FBXO31 Impairs Oocyte Quality in Premature Ovarian Insufficiency”

**DOI:** 10.14336/AD.2025.10817

**Published:** 2026-08-17

**Authors:** Feiyan Zhao, Long Yan, Xuehan Zhao, Jiaqi Wu, Ying Fang, Zhimin Xin, Hongmei Wang, Xiaokui Yang

In our article “Aberrantly High FBXO31 Impairs Oocyte Quality in Premature Ovarian Insufficiency” published in the April 2024 issue of Aging and Disease [Aging Dis.2024 Apr 1;15(2):804-823 ], we have noted some inadvertent errors. The same bright field image was incorrectly presented in Supplementary Figure 2C due to an oversight during the figure preparation process. We have attached the correct Supplementary Figure 2, ensuring the accurate depiction of the data.

**Supplementary Figure 2.**
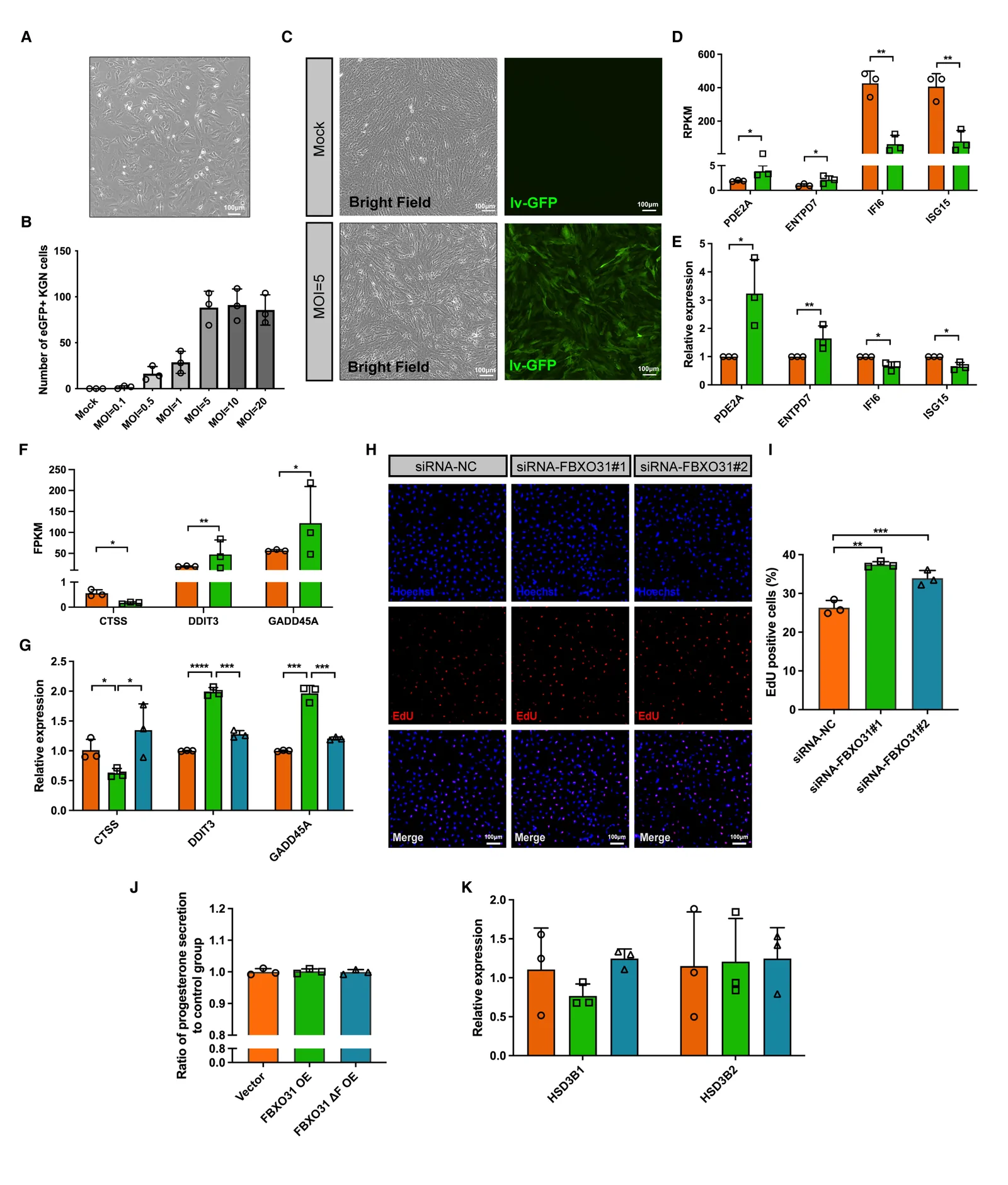
**Detection of the transfection efficiency and functional alterations in granulosa cells**. (**A**) Morphology of KGN cell lines used to establish a stable infected cell model. Scale bars: 100 μm. (**B**) Gradient MOI values used to infect KGN cells with lentivirus. (**C**) The representative images of GFP-positive cells in optimal MOI = 5. LV-GFP+, green. Scale bars: 100 μm. (**D**) The expression profiles of two upregulated genes (PDE2A and ENTPD7) and two downregulated genes (IFI6 and ISG15) from the RNAseq data. (n=3 per group) (E) The qRT-PCR validation of four DEGs in Fig. S2D. (n=3 per group, gene expressions were normalized to GAPDH) (F) The expression profiles of apoptosis-related genes from the RNA-seq data (n=3 per group). (**G**) qRT-PCR validation of apoptosis-related genes in Fig. S2F. (n=3 per group, gene expressions were normalized to GAPDH) (H) EdU staining of FBXO31 KD cells. Nuclei were stained by using Hoechst 33342. EdU positive cells, red; cell nuclei, blue. Scale bars: 100 μm. (**I**) Statistics of EdU-positive cells quantified by counting the cells with fluorescent signal using the software ImageJ. (n=3 per group) (J) Progesterone levels in the culture supernatant of FBXO31 OE cells (n=3 per group, results were represented in nanograms of estrogen per microgram protein and then normalized to the vector group). (**K**) The expression profiles steroidogenic enzymes in FBXO31 OE cells. (n=3 per group, gene expressions were normalized to GAPDH) All data were shown as mean ± SD. *p<0.05, **p<0.01, ***p<0.001, ****p<0.0001 by Mann-Whitney U test (D-G, J-K) or Chi-square test (I).

After thorough review of all relevant data, we confirm that these corrections do not impact on the study's conclusions. The scientific findings presented in the original publication remain valid and unchanged. We sincerely apologize for these errors and any inconvenience they may have caused. Upholding the highest standards of scientific integrity and transparency remains our top priority, and we have implemented additional measures to prevent similar issues in future submissions.

