# Supplementary material for: Aberrantly High FBXO31 Impairs Oocyte Quality in Premature Ovarian Insufficiency
Source: Aging Dis. 2024 Apr 1;15(2):804–23. doi: 10.14336/AD.2023.0809 (PMC10917549; doi:10.14336/AD.2023.0809)
Supplement: Supplementary file 1 [file AD-15-2-804-s.pdf]

SUPPLEMENTARY DATA

Aberrantly High FBXO31 Impairs Oocyte Quality in Premature Ovarian Insufficiency

Feiyan Zhao, Long Yan, Xuehan Zhao, Jiaqi Wu, Ying Fang, Zhimin Xin, Hongmei Wang, Xiaokui Yang

© 2023. Zhao F et al. Published online at http://www.aginganddisease.org/EN/10.14336/AD.2023.0809

SUPPLEMENTARY DATA

A

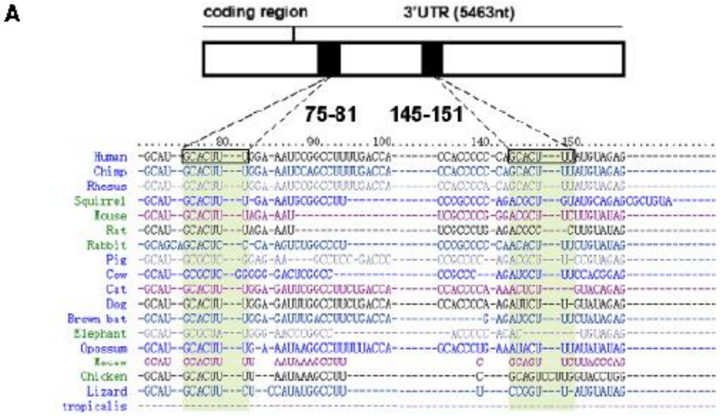

B

	Predicted consequential pairing of target region (top) and miRNA (bottom)	Site type	Context++ score	Context++ score percentage	Weighted Context++score	Conserved branch length	PCT
Position 75-81 of FBXO31 3' UTR<br>hsa-miR-106a-5p	5' ...GAAUAGAAGCAGCAUGCACUUUG...<br>||||||| 3' GAUGGACGUGACAUUCGUGAAAA	7mer-m8	-0.16	92	-0.16	6.982	0.74
Position 145-151 of FBXO31 3' UTR<br>hsa-miR-106a-5p	5' ...UCCAGCCACCCCCCAGCACUUUA...<br>||||||| 3' UAGACGUGACAGU-CGUGAAAU	8mer	-0.06	83	-0.06	0.379	< 0.1

C

FBXO31-3'UTR mut1 (75-81) 5'…gaauagaagcagcauCGUGAAAc…3'

FBXO31-3'UTR mut2 (145-151) 5'…aguccagccaccccccaCGUGAAAa…3'

FBXO31-3'UTR mut3 (75-81) (145-151) 5'…gaauagaagcagcauCGUGAAAc…aguccagccaccccccaCGUGAAAa…3'

Supplementary Figure 1. FBXO31 is a direct target of miR-106a-5p with two binding sites in the 3'UTR. (**A**) Sequence alignment of the predicted target sites in FBXO31 mRNA 3'UTR among different species. (**B**) The miR-106a-5p specific binding sites on FBXO31 predicted by TargetScan. (**C**) Sequence alignments of binding sites for miR-106a-5p on the mutated FBXO31 3′UTR.

© 2023. Zhao F et al. Published online at http://www.aginganddisease.org/EN/10.14336/AD.2023.0809

SUPPLEMENTARY DATA

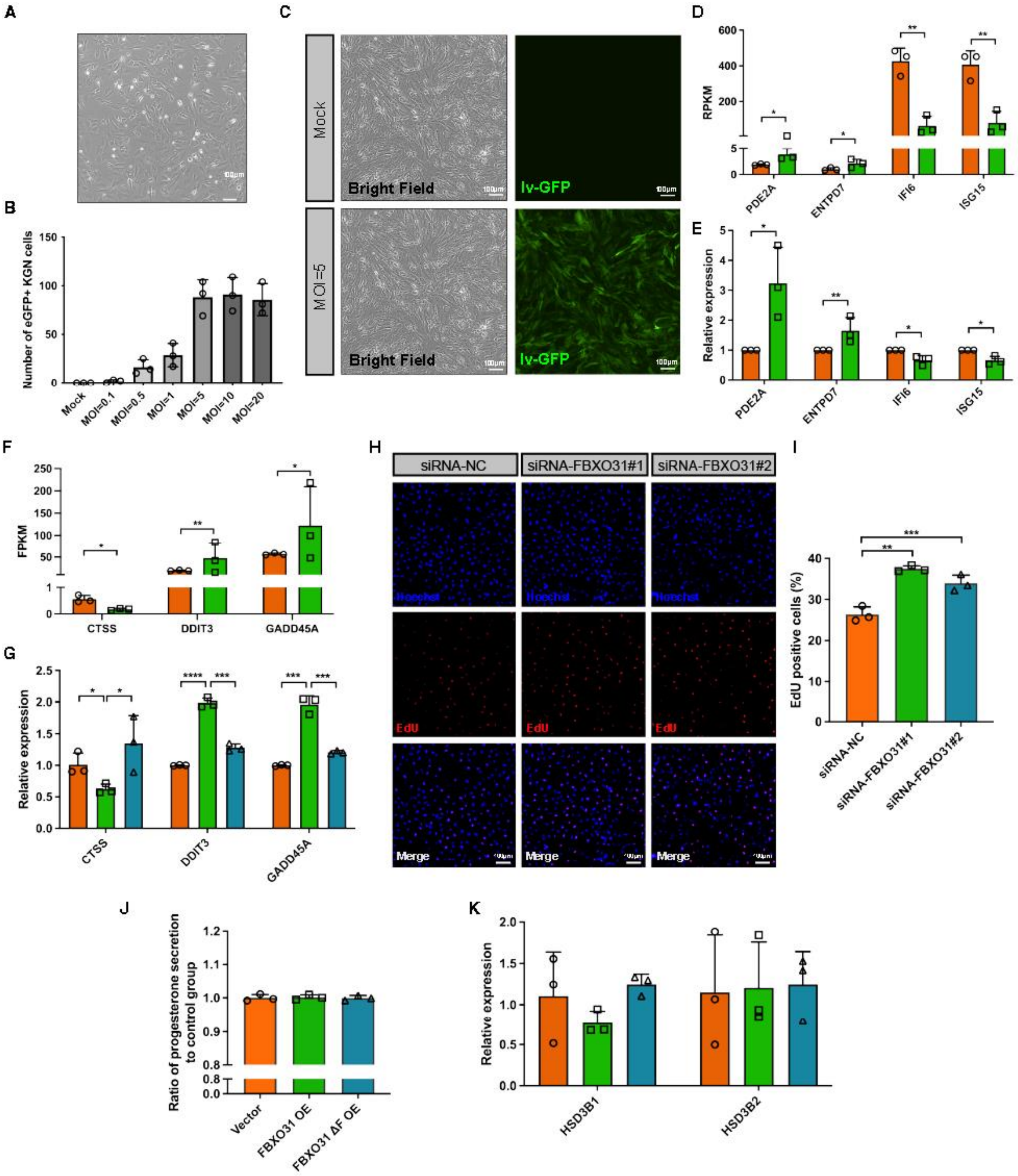

Supplementary Figure 2. Detection of the transfection efficiency and functional alterations in granulosa cells. (**A**) Morphology of KGN cell lines used to establish a stable infected cell model. Scale bars: 100 µm. (**B**) Gradient MOI values used to infect KGN cells with lentivirus. (**C**) The representative images of GFP-positive cells in optimal MOI = 5. LV-GFP+, green. Scale bars: 100 µm. (**D**) The expression profiles of two upregulated genes (PDE2A and ENTPD7) and two downregulated genes (IFI6 and ISG15) from the RNA-seq data. (n=3 per group) (**E**) The qRT-PCR validation of four DEGs in **Fig. S2D**. (n=3 per group, gene expressions were normalized to GAPDH) (**F**) The expression profiles of apoptosis-related genes from the RNA-seq data (n=3 per group). (**G**) qRT-PCR validation of apoptosis-related genes in **Fig. S2F**. (n=3 per group, gene expressions were normalized to GAPDH) (**H**) EdU staining of FBXO31 KD cells. Nuclei were stained by using Hoechst 33342. EdU positive cells, red; cell nuclei, blue. Scale bars: 100 µm. (**I**) Statistics of EdU-positive cells quantified by counting the cells with fluorescent signal using the software ImageJ. (n=3 per group) (**J**) Progesterone levels in the culture supernatant of FBXO31 OE cells (n=3 per group, results were represented in nanograms of estrogen per microgram protein and then normalized to the vector group). (**K**) The expression profiles steroidogenic enzymes in FBXO31 OE cells. (n=3 per group, gene expressions were normalized to GAPDH) All data were shown as mean ± SD. *p<0.05, **p<0.01, ***p<0.001, ****p<0.0001 by Mann-Whitney U test (D-G, J-K) or Chi-square test (I).

© 2023. Zhao F et al. Published online at http://www.aginganddisease.org/EN/10.14336/AD.2023.0809

SUPPLEMENTARY DATA

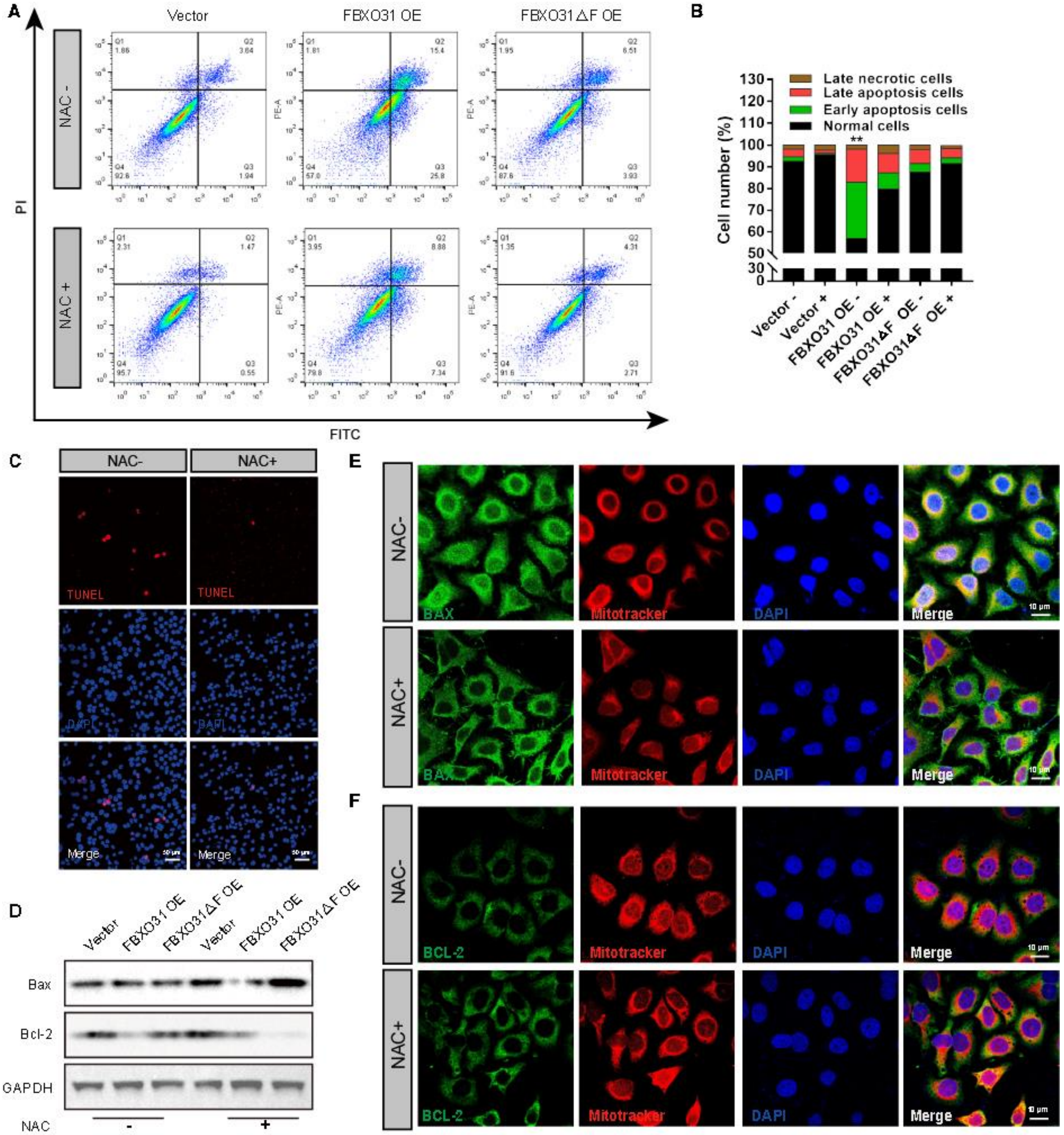

Supplementary Figure 3. Apoptosis induced by FBXO31 overexpression is mostly attributed to ROS accumulation. (**A**) Apoptosis of FBXO31 OE and FBXO31△F OE cells with or without NAC treatment detected by Annexin-V-FITC/PI staining. (**B**) Percentages of Annexin-V-FITC/PI-positive cells from gated cells. -, treatment without NAC; +, treatment with NAC. (**C**) Representative images of the apoptotic cells reflected by TUNEL staining in FBXO31 OE groups with/without NAC treatment. Scale bars: 50 µm. (**D**) WB results showing the effects of Bax and Bcl-2 in FBXO31-overexpressed cells with/without 10 mM NAC treatment. (**E,F**) Representative images of the Bax (**E**) and Bcl-2 (**F**) in FBXO31 OE cells with/without NAC treatment, mitochondria were stained with mitotracker. Scale bars: 10 µm.

© 2023. Zhao F et al. Published online at http://www.aginganddisease.org/EN/10.14336/AD.2023.0809

SUPPLEMENTARY DATA

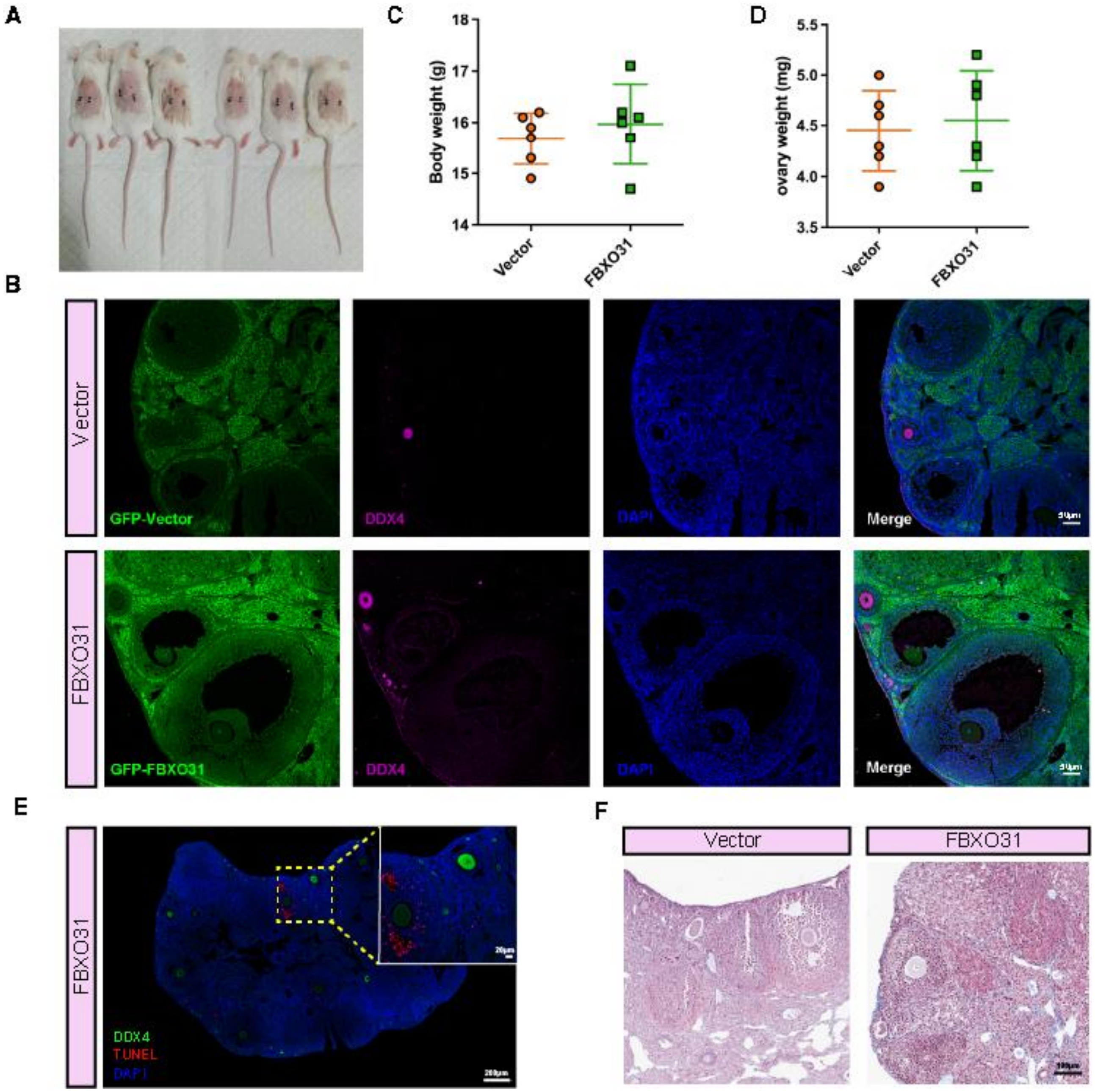

Supplementary Figure 4. Effects of FBXO31 overexpression in mouse ovaries. (**A**) Photos of mice undergoing lentivirus microinjection to the mouse ovaries. (**B**) Immunofluorescence staining of DDX4 and validation GFP expression of lentivirus in the mouse ovaries. Scale bars: 50 µm. (**C**) Body weight of mice in two groups. (n=6 per group) (**D**) Ovarian weight of mice in two groups. (n=6 per group) (**E**) TUNEL-positive cells are hardly visible in the primordial or primary follicles of ovaries microinjected with FBXO31 lentivirus. Scale bars: 200 µm (**F**) Masson trichrome staining of the ovaries from two groups. Scale bars: 100 µm. All data were reported as mean ± SD and statistically analyzed by Mann-Whitney U test (C and D).

© 2023. Zhao F et al. Published online at http://www.aginganddisease.org/EN/10.14336/AD.2023.0809

SUPPLEMENTARY DATA

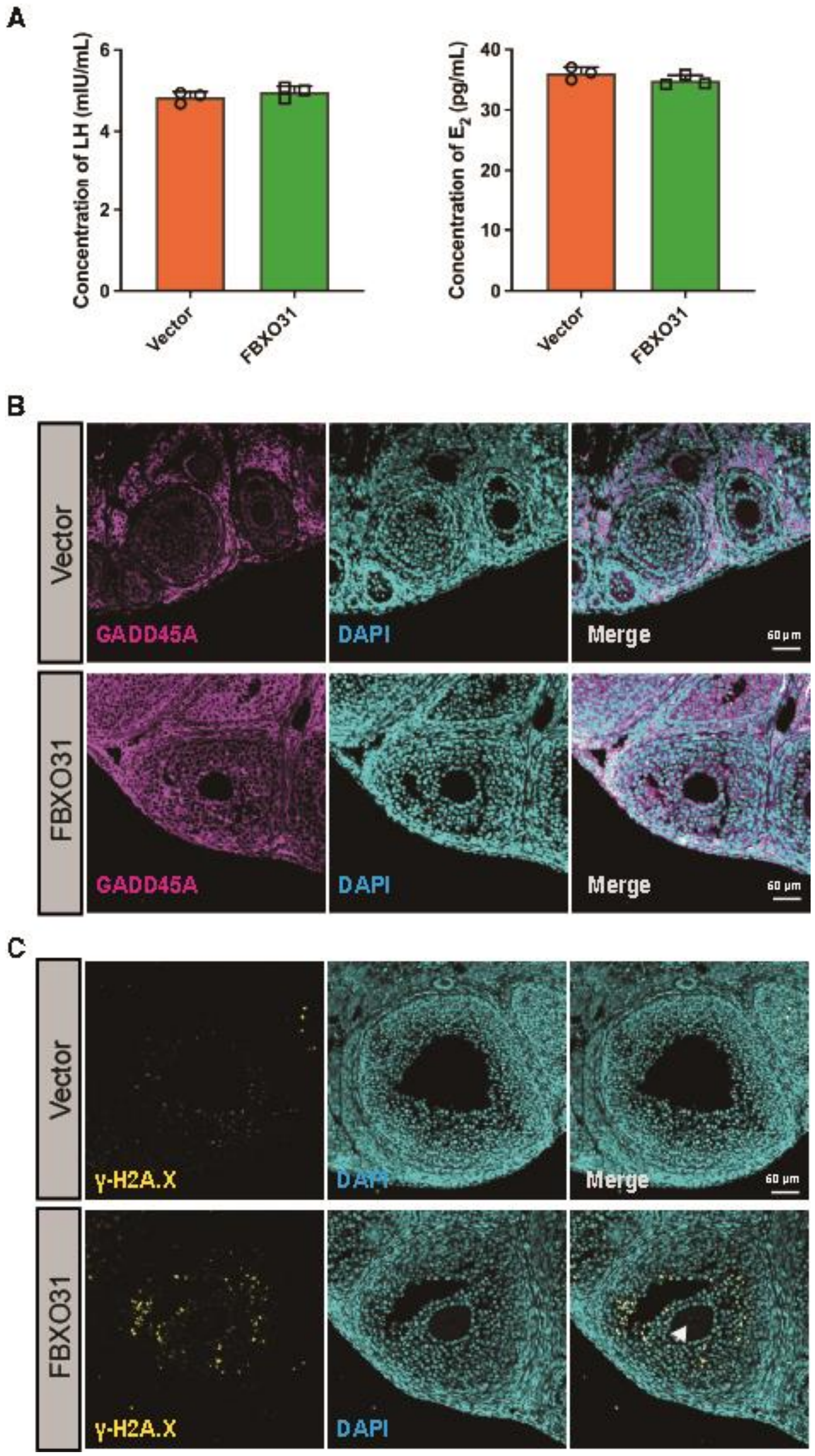

Supplementary figure 5. Effects of FBXO31 overexpression in mouse ovaries. (**A**) Effects of FBXO31 overexpression on serum E2 and LH levels detected by ELISA. (n=3 per group, data were reported as mean ± SD and statistically analyzed by Mann-Whitney U test.) (**B**) Representative images of the GADD45A levels in the mouse granulosa cells of FBXO31 and vector groups. Scale bars: 50 µm. (**C**) Representative images of γ-H2A.X in the mouse granulosa cells of FBXO31 and vector groups. White arrowheads indicated the DNA damage in the oocyte. Scale bars: 50 µm.

© 2023. Zhao F et al. Published online at http://www.aginganddisease.org/EN/10.14336/AD.2023.0809

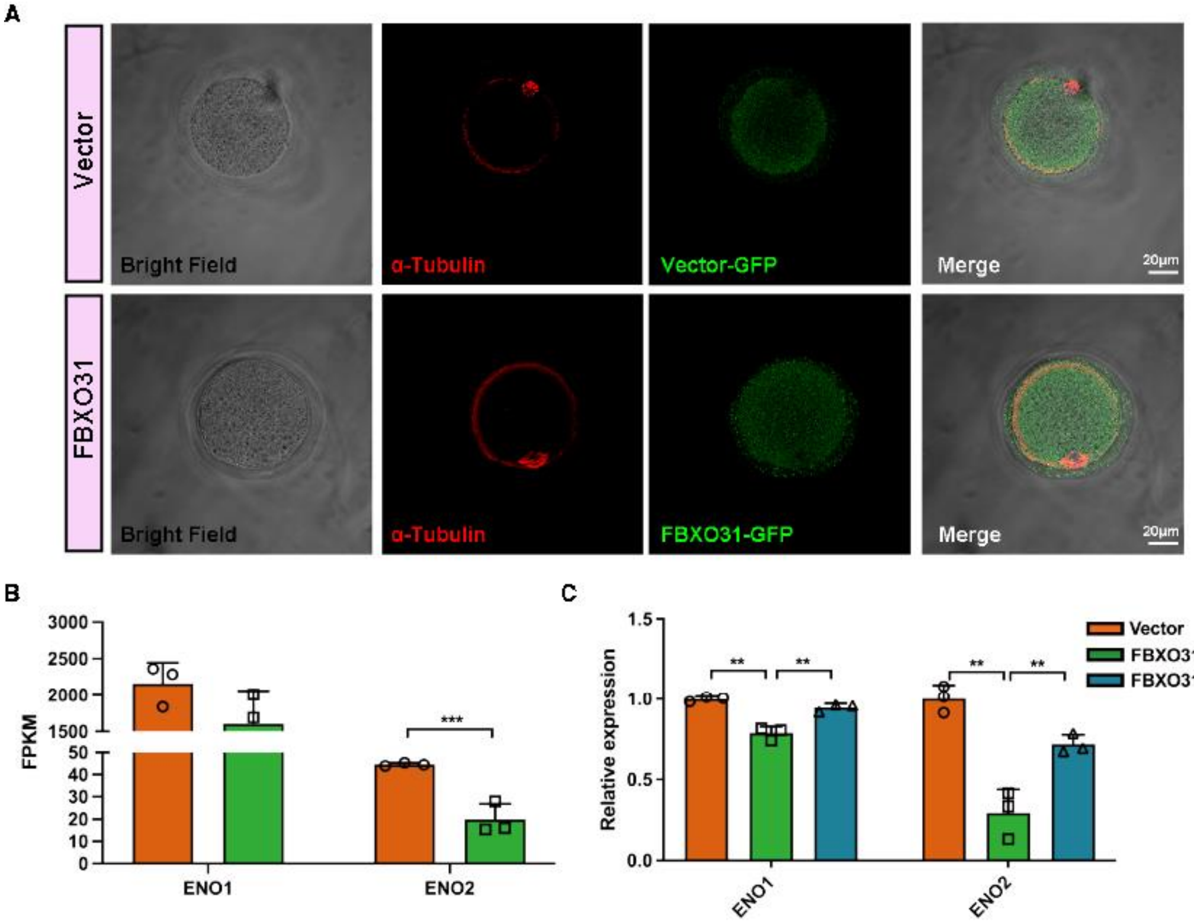

Supplementary Figure 6. Effects of FBXO31 overexpression in mouse oocytes. (**A**) Immunofluorescence staining of α-tubulin in oocytes from FBXO31 and vector groups. Scale bars: 20 μm. (**B**) The expression profiles of glycolysis-related genes from the RNA-seq data (n=3 per group). (**C**) qRT-PCR validation of the ENO1 and ENO2 in **Fig. S6B** (n=3 per group, gene expressions were normalized to GAPDH). All data were reported as mean ± SD. $**p<0.01$, $***p<0.001$ by Mann-Whitney U test (B and C).

© 2023. Zhao F et al. Published online at http://www.aginganddisease.org/EN/10.14336/AD.2023.0809

SUPPLEMENTARY DATA

Supplementary Table 1. Primers sets, related to the experimental procedures.

Gene	Primer	Sequence (5'-3')	Related Figures
miR-106a-5p	Forward	GATGCTCAAAAAGTGCTTACAGTGCA	Fig 1B
	Reverse	TATGGTTGTTCTGCTCTCTGTCTC	
U6	Forward	TGCGGGTGCTCGCTTCGGCAGC	Fig 1B
	Reverse	CCAGTGCAGGGTCCGAGGT	
FBXO31	Forward	AATCCGGCCTTTTGACCAGA	Fig 1D
	Reverse	TCCGCTCACAGGAAGAGCAC	
GAPDH	Forward	GAGTCAACGGATTTGGTCGTATTG	Fig 1D
	Reverse	CCTGGAAGATGGTGATGGGATT	
FSHR	Forward	TCTGTCACTGCTCTAACAGGG	Fig 2M
	Reverse	TGCACCTTTTTGGATGACTCG	
STAR	Forward	CCTGAGCAGAAGGGTGTCAT	Fig 2M
	Reverse	AGGACCTGGTTGATGATGCT	
CYP11A1	Forward	TGGCATCCTCTACAGACTCCTG	Fig 2M
	Reverse	CTTCAGGTTGCGTGCCATCTCA	
CYP19A1	Forward	GACGCAGGATTTCCACAGAAGAG	Fig 2M
	Reverse	ATGGTGTCAGGAGCTGCGATCA	
PDE2A	Forward	GAAAGTCCGGGAGGCTATCAT	Fig S2E
	Reverse	CACTTGGGTATCAGGAGCCA	
ENTPD7	Forward	CCCCTTTACATCCTCTGCAC	Fig S2E
	Reverse	GTCAAACTCCAACGGCAAAT	
IFI6	Forward	CTCTTCACTTGCAGTGGGGT	Fig S2E
	Reverse	TGCTGGCTACTCCTCATCCT	
ISG15	Forward	GTGGACAAATGCGACGAACC	Fig S2E
	Reverse	TCGAAGGTCAGCCAGAACAG	
CTSS	Forward	TGGATCACCACTGGCATCTCTG	Fig S2G
	Reverse	GCTCCAGGTTGTGAAGCATCAC	
DDIT3	Forward	GGTATGAGGACCTGCAAGAGGT	Fig S2G
	Reverse	CTTGTGACCTCTGCTGGTTCTG	
GADD45A	Forward	CTGGAGGAAGTGCTCAGCAAAG	Fig S2G
	Reverse	AGAGCCACATCTCTGTCGTCGT	
HSD3B1	Forward	GTCTTCGGTGTCACTCACAGAG	Fig S2K
	Reverse	CTGGTGTAGATGAAGACTGGCAC	
HSD3B2	Forward	GTCATCCACACCGCCTGTAT	Fig S2K
	Reverse	CACAGGCCTCCAACAGTAGC	
ENO1	Forward	AGTCAACCAGATTGGCTCCGTG	Fig S6B
	Reverse	CACAACCAGGTCAGCGATGAAG	
ENO2	Forward	AGCCTCTACGGGCATCTATGA	Fig S6B
	Reverse	TTCTCAGTCCCATCCAACTCC	

© 2023. Zhao F et al. Published online at http://www.aginganddisease.org/EN/10.14336/AD.2023.0809
